# Suspect and non-target screening of chemicals in clothing textiles by reversed-phase liquid chromatography/hybrid quadrupole-Orbitrap mass spectrometry

**DOI:** 10.1007/s00216-021-03766-x

**Published:** 2021-11-16

**Authors:** Josefine Carlsson, Francesco Iadaresta, Jonas Eklund, Rozanna Avagyan, Conny Östman, Ulrika Nilsson

**Affiliations:** Department of Materials and Environmental Chemistry, Arrhenius Laboratory, Stockholm University, 10691 Stockholm, Sweden

**Keywords:** Textile chemicals, Suspect screening, Non-target screening, LC/MS, High-resolution MS, Orbitrap MS

## Abstract

The global manufacturing of clothing is usually composed of multistep processes, which include a large number of chemicals. However, there is generally no information regarding the chemical content remaining in the finished clothes. Clothes in close and prolonged skin contact may thus be a significant source of daily human exposure to hazardous compounds depending on their ability to migrate from the textiles and be absorbed by the skin. In the present study, twenty-four imported garments on the Swedish market were investigated with respect to their content of organic compounds, using a screening workflow. Reversed-phase liquid chromatography coupled to electrospray ionization/high-resolution mass spectrometry was used for both suspect and non-target screening. The most frequently detected compound was benzothiazole followed by quinoline. Nitroanilines with suspected mutagenic and possible skin sensitization properties, and quinoline, a carcinogenic compound, were among the compounds occurring at the highest concentrations. In some garments, the level of quinoline was estimated to be close to or higher than 50,000 ng/g, the limit set by the REACH regulation. Other detected compounds were acridine, benzotriazoles, benzothiazoles, phthalates, nitrophenols, and organophosphates. Several of the identified compounds have log*P* and molecular weight values enabling skin uptake. This pilot study indicates which chemicals and compound classes should be prioritized for future quantitative surveys and control of the chemical content in clothing as well as research on skin transfer, skin absorption, and systemic exposure. The results also show that the current control and prevention from chemicals in imported garments on the Swedish market is insufficient.

**Graphical abstract:**

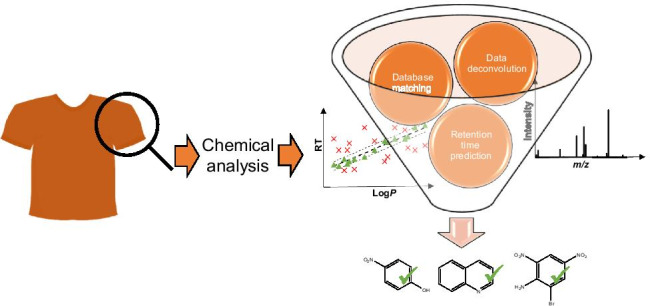

**Supplementary Information:**

The online version contains supplementary material available at 10.1007/s00216-021-03766-x.

## Introduction

Over time, the textile industry has used large amounts of numerous chemicals throughout the production chain [1]. UV stabilizers, such as benzotriazoles and benzothiazoles, are often detected in textile samples [2–5]. These chemicals are added to protect polymers against UV radiation [6] and prevent photofading as well as phototendering [7]. To increase flexibility and durability of polyvinylchloride (PVC)–based coatings, phthalates are commonly added to the textile material [8, 9]. Furthermore, dyes belonging to different chemical classes are used in the coloring step [10].

Many chemicals may also unintentionally end up in the finished textiles. Pollutants can be adsorbed from the surrounding air, and in the case of natural fibers such as cotton, it is possible to detect toxic pesticide residues that have been employed in cultivation or added for preservation during storage [11]. Other examples are aromatic amines, such as nitroanilines, of which some are used as fungicides to increase textile longevity [12], while those mostly detected in textiles are dye degradation products and/or dye impurities [13–15].

A comprehensive overview of chemicals used in textiles was published in 2012 [16], and another more recent review has focused on flame retardants, trace elements, aromatic amines, quinolines, bisphenols, benzotriazoles, benzothiazoles, and microplastics from textiles [17].

The global consumption of textiles is increasing, and the average annual textile consumption in 2017 was approximately 26 kg/EU citizen, of which clothes account for two thirds of the total consumption [18]. The two main issues associated with textile-related contamination are dermal exposure and environmental release. The former takes place by skin absorption of chemicals due to direct and prolonged contact with clothes [19, 20], while the release of chemicals from textiles during laundering is a source of pollution of the aquatic environment [4, 21].

Studies on health effects related to textile exposure have so far been mainly focused on contact allergy caused by dyes [22–24]. However, carcinogenic and mutagenic effects have also been demonstrated for a number of azo-dyes used in textile materials [25], and 22 aromatic amines that can be released from such dyes are banned at concentrations above 30,000 ng/g in textiles in Europe by the Appendix 8 of the REACH (Registration, Evaluation, Authorization and Restriction of Chemicals) regulation [26]. Triclosan, widely used as a fungicide in clothing, has been associated with endocrine disruption [27], and brominated flame retardants and phthalates, commonly used in textile production, have shown reproductive and developmental toxicities [28, 29]. A recent review has summarized different clothing-mediated exposures to chemicals and particles. Apart from exposures from newly bought clothes, it discusses exposures to compounds deposited and accumulated in the clothes from environmental sources, such as fire smoke, pesticides, nicotine, etc. [30].

As mentioned above, environmental issues may arise from chemicals released during laundering. Household laundry waste has been estimated to account for 2% of the total volume flowing in the municipal wastewater treatment plants. In a study, 72 out of 126 compounds belonging to different chemical classes were detected in both laundry wastewater and in the wastewater treatment plant effluents. Among the detected compounds, the estimated annual contribution from the washing of new clothes to the total environmental output of 4,4′-sulfonyldiphenol, phthalates, and organophosphates was considered to be substantial [21].

The large number of intermediate steps involved in textile production, together with rapid changes in the use of chemicals, caused by, e.g., fashion trends, complicates the registration process of such chemicals for importers to the EU [31]. The EU REACH regulation is the essential piece of chemical legislation within the EU, together with the Regulation on Classification, Labelling and Packaging (CLP) [32, 33]. However, REACH is not developed to take into consideration chemicals in articles such as textiles [34]. Furthermore, manufacturers and importers are obliged to register substances in quantitates exceeding 1 ton per year, but many substances are used in lower amounts in the textile production and registration is thus not required [35]. Therefore, the chemicals used may have several sources, and the information regarding those actually used in the many manufacturing processes is so far insufficient [34]. This, along with sparse analytical data, makes chemicals in textiles an important research field.

High-performance liquid chromatography (HPLC) coupled to high-resolution mass spectrometry (HRMS) plays an important role in the discovery of new contaminants. The HRMS screening methods enable the detection of a large number of compounds in a single run. Additionally, with hybrid instruments like a quadrupole-Orbitrap, it is possible to collect MS/MS data for further identification. Typically, the screening by HRMS can be divided into target, suspect, and non-target methods [14, 36, 37]. HPLC/HRMS screening of chemicals in textiles has been applied on “substances of very high concern” (SVHCs), carcinogenic dyes, and/or their isomers [38]. Ambient mass spectrometry was applied for the detection of nonylphenol ethoxylates, widely used as surfactants in textiles [39].

In this work, we present a suspect and non-target screening approach applied on twenty-four imported clothing garments on the Swedish market, characterized by different colors, origin, and materials. To our knowledge, this is the first time a screening of skin-close garments is presented with the aim to obtain an overall picture of the most frequently occurring organic compounds that could be of health concern and thus should be prioritized in further quantitative studies.

## Materials and methods

### Chemicals and solvents

Methanol, dichloromethane, ultrapure water, and formic acid were purchased from VWR Chemicals (Fontenay sous Bois, France). Acetonitrile was purchased from Rathburn Chemicals (Walkerburn, Scotland), and ammonium acetate (analytical reagent) from Riedel-de Häen (Seelze, Germany). All solvents were of HPLC grade. Clothes were purchased from four different stores in Stockholm. The samples were garments that are normally used in skin-close contact, such as t-shirts, socks, and underwear. More details regarding the samples are presented in Table 1. The standard references used for identifications are presented in Table S1, Supplementary information, SI.

**Table 1:** Sample details: color, material, garment type, and manufacturing country

Sample	Color	Material	Garment	Country
1	Pink	100% polyester	T-shirt	Vietnam
2	Pink	100% polyester	T-shirt	Bangladesh
3	Pink	100% polyester	T-shirt	China
4	Black	100% polyester	T-shirt	Vietnam
5	Black	100% polyester	T-shirt	Vietnam
6	Black	100% polyester	T-shirt	China
7	Yellow	100% polyester	T-shirt	China
8	Yellow	100% polyester	T-shirt	Bangladesh
9	Yellow	57% polyamide, 43% polyester	T-shirt	Turkey
10	Blue	90% polyester, 10% elastane	T-shirt	N.A
11	Blue	91% polyester, 9% elastane	T-shirt	Indonesia
12	Blue	90% polyester, 10% elastane	T-shirt	China
13	Red	85% polyester, 15% cotton	T-shirt	China
14	Red	87% polyester, 13% elastane	T-shirt	China
15	Red	100% polyester	Sweatshirt	N.A
16	Blue	95% cotton, 5% elastane	Underwear	Bangladesh
17	Black	85% cotton, 13% polyamide, 2% elastane	Socks	Turkey
18	White	98% polyamide, 2% elastane	Socks	N.A
19	Blue	85% cotton, 13% polyamide, 2% elastane	Socks	Turkey
20	Black	95% cotton, 5% elastane	Underwear	Bangladesh
21	Blue	95% cotton, 5% elastane	Underwear	Pakistan
22	Green	87% polyester, 13% elastane	Sweatshirt	China
23	Black	70% polyester, 25% cotton, 5% elastane	Socks	N.A
24	Purple	53% polyamide, 32% polyester, 10% elastane	Sports top	China

### Sample extraction

Textile samples were extracted by the method described by Luongo et al. [15]. Briefly, 1 g of each textile was cut into approximately 1 × 1-cm pieces and placed into a 15-mL glass test tube. A volume of 6 mL of dichloromethane was added and the sample ultra-sonicated for 10 min. The extraction was repeated once and the two extracts were pooled. A volume of 200 µL of ultrapure water was added as a keeper before evaporation at 35 °C under a gentle stream of nitrogen to a final volume of 200 µL. Subsequently, a volume of 800 µL of methanol was added and the sample extract was filtered through a 30-µm nylon membrane syringe filter (NTK Kemi, Uppsala, Sweden) into an HPLC autosampler vial. Twenty-four samples together with two procedural blanks were extracted and analyzed in duplicates using two different MS acquisition modes.

### Instrumental analysis

The samples were analyzed by HPLC coupled to a QExactive HF hybrid quadrupole-Orbitrap™, using electrospray ionization (ESI) operated both in positive and negative ion modes. Chromatographic separation was achieved on an ACE C_18_ 50 × 2.1 mm column with 3-µm particles and an ACE C_8_ guard column (Advanced Chromatographic Technologies, Aberdeen, Scotland). The flow rate was set to 0.4 mL/min and the injection volume was 5 µL. Mobile phase A consisted of 10 mM ammonium acetate, pH 7, in ultrapure water, and mobile phase B was pure acetonitrile. The gradient used was 5% B kept for 1 min, followed by a linear increase to 95% B in 20 min. The final composition was held for 5 min before returning to initial conditions, the latter held for 5 min prior to the next injection. The sheath gas flow rate was set to 30 arbitrary units (AU), the auxiliary gas flow rate to 5 AU, the spray voltage to 3.5 kV, the S-lens RF level to 55 AU, and the auxiliary gas heater to 350 °C.

In the suspect screening, an inclusion list was utilized with a combined full MS scan and data-dependent acquisition (dd-MS^2^ Top 5) in both positive and negative ion modes. When the mass analyzer detects a m/z present in the inclusion list, fragmentation is triggered. The inclusion list was created from a suspect list that consisted of 149 exact masses from SVHCs [26] listed by the Swedish Chemicals Agency [35] as well as previously detected textile compounds [3, 4, 13], SI, Table S2. The full MS scan range was set from 66.7 to 1000 m/z with a resolution of 120,000 at m/z 400. The automatic gain control (AGC) target was set to 3 × 10^6^ (number of ions), with a maximum injection time of 256 ms. For dd-MS^2^, the resolution was set to 30,000 at 400 m/z, the AGC target to 1 × 10^4^ ions, and the maximum injection time to 64 ms. The isolation window for the precursor ion selected by the quadrupole was 1.0 m/z and normalized collision energy 30 AU. The minimum AGC target was 8 × 10^3^ ions, and the chromatographic peak width was set to 30 s. Lock mass was not used, but the HRMS instrument was calibrated directly before each batch run.

For non-target screening, the same acquisition parameters and top-5 approach as for the suspect screening were used, but no inclusion list was activated. An exclusion list was compiled from multiple injections of blanks to exclude masses, based on average intensities, corresponding to instrumental noise and solvent impurities.

### Data treatment and compound identification

The software Compound Discoverer 3.0 (Thermo Fisher, MA, USA) was used to process the HRMS raw data. This software suggests possible compounds based on a workflow including matching of spectra and isotopic patterns as well as retention time alignment. The mass range was set to 100–1000 m/z, the minimum spectral intensity to 5 × 10^4^ counts, and the minimum signal-to-noise level (S/N) to 5. Retention times were aligned between samples using a mass tolerance of 5 ppm and a maximum retention time shift of 0.2 min. The maximum deviation from the theoretical relative isotope intensities was set to 30%. The software predicted molecular formulas with a mass tolerance of 5 ppm, a maximum element composition of C90 H190 Br3 Cl8 F18 N10 O18 P3 S5, a maximum H/C ratio of 3.5, and a maximum ring + double bond equivalents (RDBE) of 40. The corresponding minimum settings were CH, a H/C ratio of 0.1, and a RDBE = 0, respectively. In the case of more than one predicted molecular formula, a maximum of three formulas were searched in two databases, ChemSpider (Royal Society of Chemistry, London, England) for literature references and mzCloud (HighChem LLC, Slovakia) for matching of MS^2^ spectra. In the non-target screening, suggested formulas were kept for compounds having more than 500 references in ChemSpider, and from mzCloud, only hits with a match higher than 80% were kept.

The following described steps were applied for both suspect and non-target data treatment. The data from Compound Discoverer were exported to Microsoft Excel, and suggestions not present in duplicate, without MS^2^-data or detected with an intensity lower than 5 times the average blank peak areas (*N* = 2) were discarded.

Retention time prediction models for GC have been implemented for decades, and today, they are also common in screening studies using HPLC [40–43]. In this study, a simple linear retention time prediction model was used in order to decrease the number of false suggestions. The model was obtained by interpolating log*P* values vs retention time of 18 model compounds exhibiting log*P* values ranging from 1.3 to 8.9 (SI, S3). The predicted theoretical retention times were compared with the experimental and suggestions with retention times deviating more than ± 2 min were generally discarded.

According to the literature, a scale of identification confidence is widely adopted in non-target screening [44]. It includes 5 levels, where features belonging to level 5 have the lowest identification confidence, relying only on the measured accurate m/z. According to this scale, suggestions that have a sufficiently high MS^2^ spectrum match in mzCloud can be assigned to level 2. The suggestions from ChemSpider are of lower certainty, and other plausible compounds are possible.

Substances identified at level 1 require confirmation by use of reference compounds. In the present study, a combination of 5-ppm mass accuracy, MS^2^ fragmentation, isotopic pattern, and HPLC retention time was used for the comparison with reference compounds.

## Results and discussion

### Experimental conditions

Non-target screening can be divided into two phases, an experimental and a data treatment part, both of which affect the final results. In non-target screening, the sample preparation should be as non-selective as possible. By using dichloromethane as extraction solvent, textile chemicals with a wide range of log*P* values can be released from the garments. Of special interest are textile compounds with the highest ability to penetrate human skin, i.e., substances with molecular weight < 500 Da and log*P* 1–3 [45].

The mode of chromatography, reversed-phase HPLC using C_18_ stationary phase, was chosen to include as wide a range as possible of chemical classes. Drawbacks are that non-optimized chromatography might lead to asymmetrical peak shapes, especially for acidic/basic compounds, and also in some cases no separation of structural isomers as shown in the present work. When setting up the chromatographic conditions, the retention behavior of some of the previously known textile chemicals was considered (SI, Table S3). Some basic compounds, such as quinolines, were shown to be insufficiently retained on the C_18_ stationary phase when using formic acid in the mobile phase. In the present study, we thus selected a mobile phase at pH 7 consisting of ammonium acetate in aqueous acetonitrile.

HPLC/ESI-HRMS, in both positive and negative modes, was chosen for a broad screening, including also non-volatile compounds. To obtain a high mass accuracy in full scan with the Orbitrap, a long scan time was required, while for the MS^2^ data acquisition, the resolution was reduced in order to obtain a larger number of scans and more representative product ion spectra.

A drawback with the used non-target Top-5 approach is that identification of potentially interesting compounds in trace amounts or having low ionization efficiency might not be possible.

For suspect screening, the inclusion list was divided into a positive and a negative list based on the assumed ionization mode; for example, phenols and carboxylic acids were expected to be deprotonated in negative mode while amines, phosphates, carbonyl compounds, etc. were assumed to be mainly protonated. Compounds with possibility for both protonation and deprotonation were added to both lists. In this study, only [M + H]^+^ and [M − H]^−^ ions were considered in the inclusion list.

### From suggestion to identification

#### Data processing

Compound Discoverer gave an exhaustive list of suggested compounds. As an example, the suggestions from non-target screening in negative ion mode were approximately 200,000, and therefore, the applied filtering steps were useful. However, all filtering steps have some limitations. For instance, the number of compounds included so far in mzCloud is limited (19,500 compounds at the present time). Of the compounds included in the suspect list, 54% have MS^2^ data in mzCloud.

The suggestions from ChemSpider were based on accurate m/z from the full MS scan acquisition. The suggestions are not ranked but linked to the number of literature references, and this filtering step does not consider the importance or relevance of the compounds as textile chemicals.

The retention time model was not always accurate, and some compounds, such as UV-P, showed larger deviations than the selected limit of ± 2 min. Despite a possible loss of true-positive suggestions, this filtering step was still used to provide a higher certainty of identification.

#### Identification of compounds

The data filtering reduced the initial number of suggestions from Compound Discoverer substantially, to approximately 100 and 400 for the suspect and non-target screening, respectively (SI, Table S4, S5). A semi-quantification of compounds confirmed by reference substances was performed by using one-point calibration, and any matrix effect was not investigated. Only compounds with peak intensities 10 times higher than the noise level were quantified assuming a linear response.

Altogether, twenty compounds with possible health effects were identified and roughly quantified by using the combination of suspect and non-target screening methods.

### Suspect screening

Eleven compounds were confirmed in the suspect screening. For an additional two, the compound class was confirmed, but were assigned to level 2 due to isomer coelution or lack of reference for the specific isomer. In Table 2, their detection frequencies are reported, while the estimated concentrations are listed in Table S6.

**Table 2:** Compounds identified by suspect screening

Name	Abb	Theoretical m/z	Mass accuracy (ppm)	Formula	Specific fragments (m/z)	RT (min)	Sample no	Confidence according to Schymanski et al. [44]
Positive ion mode								
Quinoline	Q	130.0657	− 3.08	C_9_H_7_N	103.0545, 95.0493	6.17	1, 2, 3, 4, 5, 6, 7, 8, 9, 10, 11, 12, 13, 14, 15, 20, 22, 23, 24	Level 1
Benzothiazole	BT	136.0221	− 3.68	C_7_H_5_NS	109.0107, 65.0388	6.32	1, 2, 3, 4, 5, 6, 7, 8, 9, 10, 11, 12, 13, 14, 15, 16, 17, 20, 21, 22, 23, 24	Level 1
Methylbenzotriazole	TTri	134.0718	− 2.98	C_7_H_7_N_3_	95.0492, 105.0448	4.86	7, 9, 22, 24	Level 2*
2-(Benzotriazol-2-yl)-4-methylphenol	UV-P	226.0980	0.00	C_13_H_11_N_3_O	79.0543, 120.0557	13.36	1, 9	Level 1
2-(2H-Benzotriazol-2-yl)-4,6-bis(1-methyl-1-phenylethyl) phenol	UV-234	448.2389	− 2.01	C_30_H_29_N_3_O	370.1913, 119.0856	19.28	18	Level 1
Methylquinoline	MQ	144.0813	− 3.47	C_10_H_9_N	115.0544, 91.0543	6.94	3, 4, 6, 11, 12, 13, 14, 15, 20, 22, 23, 24	Level 2*
Diisobutyl phthalate	DiBP	279.1597	− 3.94	C_16_H_22_O_4_	149.0234, 121.0282	13.77	22, 24	Level 1
Negative ion mode								
2-Mercaptobenzothiazole	MBT	165.9785	− 1.81	C_7_H_5_NS_2_	134.0060, 57.9755	5.82	10	Level 1
2-Bromo-4,6-dinitroaniline	2-Br-4,6-DNA	259.9307	2.31	C_6_H_4_BrN_3_O_4_	229.9331, 199.9349, 78.9190	8.65	1, 2, 3, 4, 5, 6, 10, 11, 12, 15, 20, 21, 23, 24	Level 1
2,6-Dichloro-4-nitroaniline	2,6-DCl-4-NA	204.9572	− 0.98	C_6_H_4_Cl_2_N_2_O_2_	174.9588, 59.0136	9.28	2, 4, 6, 10, 11, 12, 14, 15, 21, 23, 24	Level 1
6-Chloro-2,4-dinitroaniline	6-Cl-2,4-DNA	215.9812	0.46	C_6_H_4_ClN_3_O_4_	185.9828, 155.9848	8.25	1, 2, 3, 4, 5, 6, 8, 10, 11, 12, 15, 20, 21, 22, 23, 24	Level 1
2,4-Dinitroaniline	2,4-DNA	182.0202	− 0.55	C_6_H_5_N_3_O_4_	152.0218, 122.0236	6.95	1, 2, 3, 4, 5, 6, 10, 11, 12, 14, 15, 20, 21, 22, 23, 24	Level 1
Chloronitroaniline	Cl-NA	170.9961	− 1.17	C_6_H_5_ClN_2_O_2_	135.0190, 140.9978	7.66	4, 6, 11, 12, 13, 14, 15, 23, 24	Level 2*

*Assigned as level 2 due to isomer coelution and/or lack of reference compounds.

#### Benzothiazoles and benzotriazoles

Benzothiazole (BTs) and benzotriazole derivatives (BTris) are widely used industrial chemicals with many areas of application (e.g., plasticizers, UV stabilizers), and a number of them are associated with biological effects such as genotoxicity, skin irritation, skin sensitization, and toxicity to aquatic species [46, 47].

BT, the most prevalent detected compound, was identified in 22 of the 24 investigated clothes with concentrations up to 230 ng/g textile. In the literature, BT is frequently reported in textile materials within a similar concentration range [3–5]. In four samples, a peak matching 5-methylbenzotriazole (5-TTri) was detected. Since more isomeric forms are possible, it was just assigned as a methylbenzotriazole (TTri). UV-P was identified in two samples, despite a large deviation from the predicted retention time, and UV-234 was identified in one of the samples. The estimated concentrations for TTri (≤ 8 ng/g) and UV-234 (200 ng/g) were within previously reported, while the levels of UV-P (470 and 800 ng/g) were approximately 10 times higher [2]. Mercaptobenzothiazole (MBT) was identified in one sample, a blue T-shirt made of blended polyester and elastane.

#### Quinolines

Quinoline, used in dye manufacturing, is a carcinogen and suspected mutagen and thus regulated in textiles by REACH to 50,000 ng/g [26]. In the present study, quinoline and methylquinolines were the second most frequently detected compounds, being detected in nineteen and twelve garments, respectively. Several methylquinoline isomers were detected, but due to isomer coelution, they were quantified together as a group by assuming identical response factors. Quinolines were measured at highly varying concentrations up to 60,000 ng/g, which are consistent with values previously reported by our research group [4, 13, 15]. In some garments, the level of quinoline was close to or above the 50,000 ng/g limit.

#### Phthalates

Phthalates are used in the textile production as plasticizers. Diisobutyl phthalate (DiBP), among other phthalates, is included in the REACH candidate list of SVHCs due to reprotoxic and endocrine-disrupting properties, and toxicity to aquatic life [26]. In this work, DiBP was detected in two samples, with the highest estimated concentration at 3300 ng/g. Phthalates have previously been reported at concentration levels > 0.1% (w/w) in textiles and up to 20% (w/w) in PVC coating patterns [8, 48].

#### Nitroanilines

Nitroanilines belong to the class of aromatic amines and are both precursors and degradation products of dyes, as well as used as biocides [12]. Among this large group of compounds, several are biologically active, for instance with mutagenic, carcinogenic, or skin allergenic effects [49]. Dermal absorption of aromatic amines seems to be important, and their release from clothes could be a source of hazardous human exposure [50]. The use of dyes that can release one or more of 22 aromatic amines classified as carcinogens at levels exceeding 30,000 ng/g in textiles is restricted by Appendix 8 of REACH [26]. In the present study, the most prevalent detected aromatic amines were derivatives of dinitroaniline (DNA), namely 6-Cl-2,4-DNA and 2,4-DNA, each being detected in sixteen of the investigated garments, with concentrations up to 42,000 and 5900 ng/g, respectively. Two other halogenated DNAs, 2-Br-4,6-DNA (≤ 26,000 ng/g) and 2,6-DCl-4-NA (≤ 6800 ng/g) were detected in fourteen and eleven samples, respectively. Luongo et al. have previously reported similar levels of these compounds in clothes [13]. An isomer of chloronitroaniline was also identified at levels up to 165,000 ng/g.

### Non-target screening

Seven unknowns were identified in the non-target screening as shown in Table 3. Estimated concentrations are found in Table S7.

**Table 3:** Compounds identified by non-target screening

Name	Abb	Theoretical m/z	Mass accuracy (ppm)	Formula	Specific fragments (m/z)	RT, (min)	Sample no	Confidence according to Schymanski et al. [44]
Positive ion mode								
Triphenyl phosphate	TPhP	327.0786	− 2.75	C_18_H_15_O_4_P	233.0357, 153.0697	12.4	11, 17, 20, 22, 24	Level 1
Tributyl phosphate	TBP	267.1725	− 2.99	C_12_H_27_O_4_P	98.9844, 140.0106	12.3	12	Level 1
Acridine	Acr	180.0813	− 2.78	C_13_H_9_N	152.0618	8.9	3, 12, 13, 14, 24	Level 1
Negative ion mode								
2,4-Dinitrophenol	2,4-DNP	183.0041	− 1.09	C_6_H_4_N_2_O_5_	95.0140, 123.0079	3.1	4, 15, 22	Level 1
4-Nitrophenol	4-NP	138.0191	− 2.17	C_6_H_5_NO_3_	108.0211, 95.0140	4.6	1, 12, 13, 14, 15, 20, 22, 23, 24	Level 1
3-Nitrophenol	3-NP	138.0191	− 2.17	C_6_H_5_NO_3_	108.0211, 80.0268	5.8	4, 6, 7, 8, 15	Level 1
Chloronitrophenol	Cl-NP	171.9801	− 1.74	C_6_H_4_ClNO_3_	68.9983, 141.9819	3.2	12, 13, 15	Level 2*

*Assigned as level 2 due to isomer coelution and/or lack of reference compounds.

#### Nitrophenols

Nitrophenols (NPs) are industrial chemicals used in many applications, such as the manufacturing of dyes, herbicides, plastics, and pesticides. Even though NPs are extensively used in the textile industry, their occurrence in textile material seems to be poorly investigated, in spite of some being classified as toxic, persistent, and bioaccumulative [51]. They are common environmental pollutants, and the US Environmental Protection Agency has included 4-NP and 2,4-DNP in their priority list [52, 53]. To the best of our knowledge, neither concentration nor detection frequency in clothes has been reported earlier. Several compounds belonging to this chemical class were identified in the investigated garments in negative ionization mode. Among the NPs, 4-NP was the most frequently detected, in nine out of twenty-four clothes. Also, 3-NP and 2,4-DNP were detected in 5 and 3 garments, respectively. The sum of NPs was detected up to 85 ng/g, with 4-NP at the highest concentrations.

**Fig. 1:**
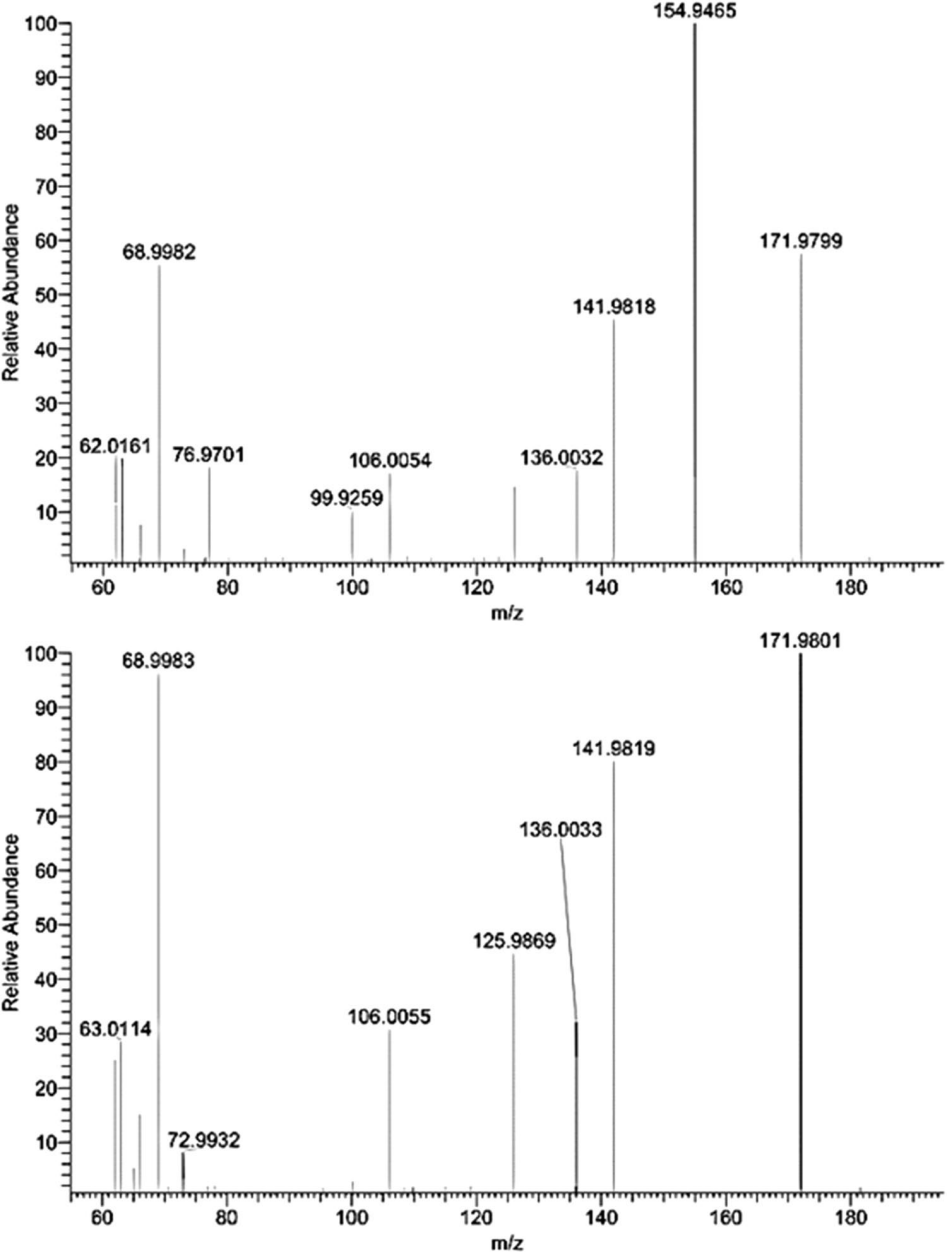
MS2 spectrum in negative ESI mode for the tentatively identified chloronitrophenol in sample 15 (top) and MS2 spectrum of the standard reference 2-chloro-4-nitrophenol (bottom). For interpretation, see section “Nitrophenols”

Another NP, chloronitrophenol, was also detected (Fig. 1), exhibiting typical radical losses in negative mode, such as NO (to m/z 141.9818), NO_2_ (to m/z 125.9869), and Cl (to m/z 136.0032). The exact mass and isotopic and fragmentation patterns matched with 2-chloro-4-nitrophenol (2-Cl-4-NP), but the retention time in the sample differed 0.7 min from the reference standard. Furthermore, as seen in the top spectrum of Fig. 1, an extra peak corresponding to m/z 154.9465 was observed in the sample but not in the reference standard. This fragment could be explained by a coeluting compound, but this was not further investigated (Fig. 1).

#### Organophosphates

The use of organophosphates as flame retardants has been considered an alternative to the bioaccumulative, persistent, and toxic polybrominated diphenyl ethers [54, 55]. Organophosphates are detected in several indoor environments, and their dermal adsorption has been suggested as an important route to human exposure [54, 56]. Even though they are considered as a safer alternative, there are a number of studies considering the health effects related to human exposure to these chemicals [57–59].

Triphenyl phosphate (TPhP) and tributyl phosphate (TBP) were identified in the investigated clothes. TBP was present in one of the samples and TPhP was identified in five samples. In this study, the highest estimated concentrations were 44,000 ng/g for TPhP and 520 ng/g for TBP. Both TBP and TPhP have previously been reported to occur in textile materials (curtains) at levels similar to our results [60].

#### Acridine

Acridine is used in the textile production as a component of several dyes, e.g., acridine orange. Acridine is a skin irritant and shows similar toxic effects as quinoline [61, 62]. To the best of our knowledge, the occurrence of acridine in textile materials has not been reported previously. In the present study, acridine was detected in five of the clothes at levels up to 940 ng/g.

## Conclusions

In the present study, screening strategies were applied to identify suspect as well as a priori unknown compounds in clothes newly bought on the Swedish market. We identified twenty compounds with various health effects, and to our knowledge, nitrophenols, organophosphates, and acridine were detected for the first time in skin-close garments. Benzothiazoles, benzotriazoles, nitroanilines, and quinolines were also identified, and their estimated concentrations were in agreement with previous results from target analysis of textile materials by our research group. The screening also detected several hundreds of tentatively identified compounds that need to be further investigated. The detected chemicals are not covalently bound to the fibers, thus possessing sufficient mobility to migrate to the human skin or to the environment. Several of the confirmed as well as tentatively identified compounds have log*P* and molecular weight values enabling skin uptake. For these reasons, these compounds are relevant considering both dermal uptake and environmental release.

The presented results can be used to obtain a better understanding of what chemicals of health relevance we are exposed to through daily textile contact, as well as improving risk assessments with respect to chemicals present in textiles. The results also point out a number of chemicals that will be prioritized for future quantitative surveys as well as research on skin uptake and systemic exposure. It also shows that the current control and prevention from chemicals in imported garments on the Swedish market is insufficient.

## Supplementary Information

Below is the link to the electronic supplementary material.

Supplementary file1 (DOCX 2377 KB)

Supplementary file2 (XLSX 58 KB)
