# Supplementary material for: Suspect and non-target screening of chemicals in clothing textiles by reversed-phase liquid chromatography/hybrid quadrupole-Orbitrap mass spectrometry
Source: Anal Bioanal Chem. 2021 Nov 16;414(3):1403–13. doi: 10.1007/s00216-021-03766-x (PMC8724091; doi:10.1007/s00216-021-03766-x)
Supplement: Supplementary file 1 — Supplementary file1 (DOCX 2377 KB) [file 216_2021_3766_MOESM1_ESM.docx]

**Suspect and Non-target Screening of Chemicals in Clothing Textiles by Reversed-Phase Liquid Chromatography–Hybrid Quadrupole-Orbitrap Mass Spectrometry**

Josefine Carlsson†, Francesco Iadaresta†, Jonas Eklund, Rozanna Avagyan, Conny Östman, Ulrika Nilsson*.

Department of Materials and Environmental Chemistry

Arrhenius Laboratory, Stockholm University
SE-10691 Stockholm, Sweden

* Corresponding author:
Email: [u](mailto:u)lrika.nilsson@mmk.su.se

Phone: +46-(0)8-162327

†Shared first authorship

Electronic Supporting Materials (ESM)

## Table S1. Standard compounds, their abbreviations, CAS-No. and supplier.

| **Compound** | **CAS-No** | **Purity** | **Supplier** | **City, Country** |
| --- | --- | --- | --- | --- |
| 1-H-benzotriazole | 95-14-7 | 99% | Sigma Aldrich | St Louis, MO, USA |
| 2-(2H-benzotriazol-2-yl)-4,6-bis(1-methyl-1-phenylethyl) phenol | 70321-86-7 | Powder | Sigma Aldrich | St Louis, MO, USA |
| 2-(2H-benzotriazol-2-yl)-4,6-di-tert-pentylphenol | 25973-55-1 | 98% | Sigma Aldrich | St Louis, MO, USA |
| 2-(benzotriazol-2-yl)-4-methylphenol | 2440-22-4 | 97% | Sigma Aldrich | St Louis, MO, USA |
| 2,2′-dithiobisbenzothiazole | 414-438-3850 | 99% | Sigma Aldrich | St Louis, MO, USA |
| 2,4-dinitroaniline | 97-02-9 | 98% | Sigma Aldrich | St Louis, MO, USA |
| 2,4-dinitrophenol | 51-28-5 | Analytical standard | Sigma Aldrich | St Louis, MO, USA |
| 2,4-di-*tert*-butyl-6-(5-chlorobenzotriazol-2-yl)phenol | 3864-99-1 | 98% | Sigma Aldrich | St Louis, MO, USA |
| 2,6-dichloro-4-nitroaniline | 99-30-9 | 96% | Sigma Aldrich | St Louis, MO, USA |
| 2-bromo-4,6-dinitroaniline | 1818-73-8 | 94% | Sigma Aldrich | St Louis, MO, USA |
| 2-mercaptobenzothiazole | 149-30-4 | ≥99% | Fluka | St Louis, MO, USA |
| 2-methylbenzothiazole | 120-75-2 | 99% | Sigma Aldrich | St Louis, MO, USA |
| 2-methylthiobenzothiazole | 615-22-5 | 97% | Sigma Aldrich | St Louis, MO, USA |
| 3-nitrophenol | 554-84-7 | ≥98% | Merck | Darmstadt, Germany |
| 4-nitrophenol | 100-02-7 | ≥98% | Merck | Darmstadt, Germany |
| 5,6-dimethylbenzotriazole | 4184-79-6 | 99% | Sigma Aldrich | St Louis, MO, USA |
| 5-methylbenzotriazole | 136-85-6 | 99% | Sigma Aldrich | St Louis, MO, USA |
| 6-chloro-2,4-dinitroaniline | 3531-19-9 | 97% | Sigma Aldrich | St Louis, MO, USA |
| 2-chloro-4-nitroaniline | 121-87-9 | 99% | Sigma Aldrich | St Louis, MO, USA |
| Acridine | 260-94-6 | 97% | Sigma Aldrich | St Louis, MO, USA |
| Benzothiazole | 95-16-9 | 96% | Sigma Aldrich | St Louis, MO, USA |
| Diisobutyl phthalate | 84-69-5 | 99% | Sigma Aldrich | St Louis, MO, USA |
| 2-methylquinoline | 91-63-4 | ≥95% | Sigma Aldrich | St Louis, MO, USA |
| 3-methylquinoline | 612-58-8 | 99% | Sigma Aldrich | St Louis, MO, USA |
| 4-methylquinoline | 491-35-0 | ≥99% | SAFC | St Louis, MO, USA |
| 6-methylquinoline | 91-62-3 | 98% | Sigma Aldrich | St Louis, MO, USA |
| 8-methylquinoline | 611-32-5 | 97% | Sigma Aldrich | St Louis, MO, USA |
| N-cyclohexyl-2-benzothiazolesulphenamide | 95-31-8 |  | Sigma Aldrich | St Louis, MO, USA |
| Quinoline | 91-22-5 | >97% | Merck | Darmstadt, Germany |
| Tributyl phosphate | 126-73-8 | ≥99% | Sigma Aldrich | St Louis, MO, USA |
| Triphenyl phosphate | 115-86-6 | Synthesis grade | Merck | Darmstadt, Germany |
| 2-chloro-4-nitrophenol | 619-08-9 | 97% | Sigma Aldrich | St Louis, MO, USA |

## Table S2 see separate Excel file

## S3. Retention time prediction model


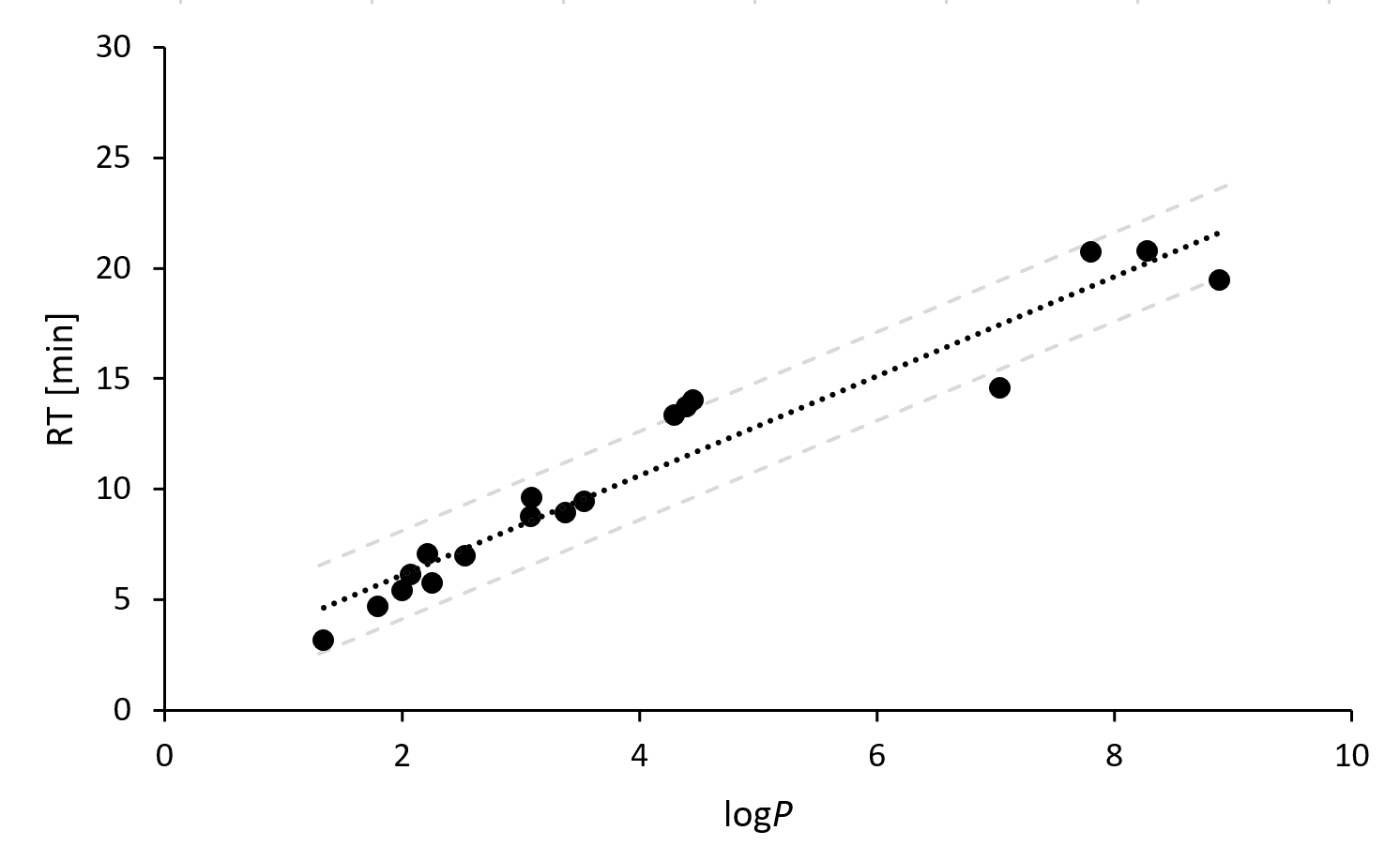


**Figure S3.** Retention time prediction model used in the non-target/suspect screening. Prediction equation: y = 2.2502x + 1.6051, R^2^=0.9306.

**Table S3**. Compounds included in the retention time prediction model, abbreviation, log*P*, experimental and predicted retention time, RT

| **Name** | **Abbreviation** | **log*P^*^*** | **Experimental RT [min]** | **Predicted RT [min]** |
| --- | --- | --- | --- | --- |
| 1-H-benzotriazole | BTri | 1.34 | 3.1 | 4.6 |
| 5-Methyl-1H-benzotriazole | 5-TTri | 1.80 | 4.7 | 5.7 |
| Benzothiazole | BT | 2.01 | 5.4 | 6.1 |
| 5,6-Dimethyl-1H-benzotriazole | XTri | 2.26 | 5.7 | 6.7 |
| 2-methylthiobenzothiazole | MTBT | 3.10 | 9.6 | 8.6 |
| 2-(benzotriazol-2-yl)-4-methylphenol | UV-P | 4.30 | 13.3 | 11.3 |
| N-cyclohexyl-2-benzothiazolesulphenamide | CBS | 4.40 | 13.7 | 11.5 |
| 2,2'-dithiobisbenzothiazole | MBTS | 7.04 | 14.6 | 17.4 |
| 2-(2H-benzotriazol-2-yl)-4,6-di-tert-pentylphenol | UV-328 | 8.28 | 20.8 | 20.2 |
| 2,4-di-tert-butyl-6-(5-chlorobenzotriazol-2-yl)phenol | UV-327 | 7.81 | 20.7 | 19.2 |
| 2-(2H-benzotriazol-2-yl)-4,6-bis(1-methyl-1-phenylethyl)phenol | UV-234 | 8.89 | 19.4 | 21.6 |
| Diisobutyl phthalate | DiBP | 4.46 | 14.0 | 11.6 |
| Quinoline | Q | 2.08 | 6.1 | 6.3 |
| Methylquinoline | Me-Q | 2.54 | 7.0 | 7.3 |
| 2-bromo-4,6-dinitroaniline | 2-Br-4,6-DNA | 3.09 | 8.7 | 8.6 |
| 2,6-dichloro-4-nitroaniline | 2,6-DCl-4-NA | 3.54 | 9.4 | 9.6 |
| 6-chloro-2,4-dinitroaniline | 6-Cl-2,4-DNA | 3.38 | 8.9 | 9.2 |
| 2,4-dinitroaniline | 2,4-DNA | 2.22 | 7.0 | 6.6 |

*Calculated with ChemOffice20 (PerkinElmer Informatics)

**Table S4** and **Table S5** see separate Excel file.

## Table S6. Estimated mean concentration of suspects ng/g (RSD%)

| **Sample** | **Me-Q** | **Q** | **BT** | **MBT** | **TTri** | **UV-P** | **UV-234** | **2-Br-4,6-DNA** | **2,6-DCl-4-NA** | **6-Cl-2,4-DNA** | **2,4-DNA** | **x-Cl-x-NA** | **DiBP** |
| --- | --- | --- | --- | --- | --- | --- | --- | --- | --- | --- | --- | --- | --- |
| 1 | N.D. | 12 (141.4) | 32 (24) | N.D. | N.D. | 817 (22.8) | N.D. | 344 (30.5) | N.D. | 21 (141.4) | 95 (6.7) | N.D. | N.D. |
| 2 | N.D. | 165 (10.1) | 20 (1.2) | N.D. | N.D. | N.D. | N.D. | 104 (10.3) | 20 (4.5) | 627 (4.8) | 61 (8.3) | N.D. | N.D. |
| 3 | 750 (0.8) | 963 (1.6) | 38 (6.7) | N.D. | N.D. | N.D. | N.D. | 23 (4.5) | N.D. | 52 (141.4) | 17 (6.3) | N.D. | N.D. |
| 4 | 39 665 (1.9) | 36 513 (4.4) | 106 (5.3) | N.D. | N.D. | N.D. | N.D. | 18 882 (1.6) | 145 (6.8) | 7 261 (2.6) | 5 960 (3.1) | 2 165 (2.3) | N.D. |
| 5 | N.D. | 470 (2.3) | 32 (5.3) | N.D. | N.D. | N.D. | N.D. | 25 755 (4.7) | N.D. | 895 (1.7) | 916 (7.6) | N.D | N.D. |
| 6 | 4 898 (1.5) | 5 571 (0.9) | 23 (5.5) | N.D. | N.D. | N.D. | N.D. | 8 192 (0.5) | 1 602 (5.5) | 42 026 (0.7) | 4 212 (0.8) | 21 396 (8.7) | N.D. |
| 7 | N.D. | 133 (0.9) | 71 (1.2) | N.D. | 1 (6.3) | N.D. | N.D. | N.D. | N.D. | N.D. | N.D. | N.D. | N.D. |
| 8 | N.D. | 51 (4.8) | 73 (1.2) | N.D. | N.D. | N.D. | N.D. | N.D. | N.D. | 106 (7.3) | N.D. | N.D. | N.D. |
| 9 | N.D. | 59 (1.7) | 75 (1) | N.D. | 8 (2.1) | 468 (141.4) | N.D. | N.D. | N.D. | N.D. | N.D. | N.D. | N.D. |
| 10 | N.D. | 158 (0.9) | 55 (0.8) | 2100 (0.08) | N.D. | N.D. | N.D. | 3 357 (3.6) | 34 (2.9) | 10 096 (2.3) | 1 133 (1.2) | N.D. | N.D. |
| 11 | 912 (1) | 1 491 (0.8) | 9 (0.4) | N.D. | N.D. | N.D. | N.D. | 20 777 (7) | 415 (8.1) | 398 (11.6) | 545 (9.1) | 7 395 (7.8) | N.D. |
| 12 | 21 115 (0.3) | 16 557 (4.3) | 13 (4.1) | N.D. | N.D. | N.D. | N.D. | 9 712 (8.3) | 6 863 (3.4) | 1 248 (5.1) | 315 (86.9) | 85 530 (6.1) | N.D. |
| 13 | 20 429 (3) | 16 173 (0.3) | 23 (97.7) | N.D. | N.D. | N.D. | N.D. | N.D. | N.D. | N.D. | N.D. | 165 222 (5.2) | N.D. |
| 14 | 58 932 (2.2) | 66 276 (2.6) | 44 (8.5) | N.D. | N.D. | N.D. | N.D. | N.D. | 1 037 (4.3) | N.D. | 122 (7) | 53 987 (5.5) | N.D. |
| 15 | 365 (4.9) | 707 (0.5) | 231 (1.5) | N.D. | N.D. | N.D. | N.D. | 250 (6) | 2 193 (0.2) | 1 194 (4.8) | 496 (3.6) | 154 766 (2.9) | N.D. |
| 16 | N.D. | N.D. | 14 (1) | N.D. | N.D. | N.D. | N.D. | N.D. | N.D. | N.D. | N.D. | N.D. | N.D. |
| 17 | N.D. | N.D. | 2 (6.2) | N.D. | N.D. | N.D. | N.D. | N.D. | N.D. | N.D. | N.D. | N.D. | N.D. |
| 18 | N.D. | N.D. | N.D. | N.D. | N.D. | N.D. | 222 (2) | N.D. | N.D. | N.D. | N.D. | N.D. | N.D. |
| 19 | N.D. | N.D. | N.D. | N.D. | N.D. | N.D. | N.D. | N.D. | N.D. | N.D. | N.D. | N.D. | N.D. |
| 20 | 528 (2.5) | 592 (4.6) | 13 (5.4) | N.D. | N.D. | N.D. | N.D. | 229 (0.7) | N.D. | 613 (6.3) | 100 (2.8) | N.D. | N.D. |
| 21 | N.D. | N.D | 3 (2.3) | N.D. | N.D. | N.D. | N.D. | 255 (3.2) | 45 (5.4) | 242 (0.3) | 43 (7.3) | N.D. | N.D. |
| 22 | 834 (1.6) | 1 330 (1.1) | 170 (1.5) | N.D. | 1 (6.4) | N.D. | N.D. | N.D. | N.D. | 39 (10) | 32 (13.6) | N.D. | 3 299 (0.5) |
| 23 | 9 531 (2) | 9 282 (0.6) | 14 (3.3) | N.D. | N.D. | N.D. | N.D. | 57 (10.7) | 5 452 (18.5) | 6 (141.4) | 5 (141.4) | 22 168 (141.4) | N.D. |
| 24 | 533 (5.1) | 300 (8.1) | 32 (3.5) | N.D. | 3 (1.7) | N.D. | N.D. | 203 (7.2) | 912 (32.8) | 1 940 (5.5) | 138 (86.6) | 85 394 (5.3) | 1 851 (3.8) |

N.D. Not Detected

## Table S7. Estimated mean concentration in non-target screening ng/g (RSD%)

| **Sample** | **TPhP** | **TBP** | **Acridine** | **2,4-DNP** | **4-NP** | **3-NP** | **x-Cl-y-NP** |
| --- | --- | --- | --- | --- | --- | --- | --- |
| 1 | N.D. | N.D. | N.D. | N.D. | 21.49 (33) | N.D. | N.D. |
| 2 | N.D. | N.D. | N.D. | N.D. | N.D. | N.D. | N.D. |
| 3 | N.D. | N.D. | 25.85 (4.7) | N.D. | N.D. | N.D. | N.D. |
| 4 | N.D. | N.D. | N.D. | 18.28 (6.7) | N.D. | 1.59 (3.4) | N.D. |
| 5 | N.D. | N.D. | N.D. | N.D. | N.D. | N.D. | N.D. |
| 6 | N.D. | N.D. | N.D. | N.D. | N.D. | 2.02 (4.3) | N.D. |
| 7 | N.D. | N.D. | N.D. | N.D. | N.D. | 17.61 (3.7) | N.D. |
| 8 | N.D. | N.D. | N.D. | N.D. | N.D. | 23.93 (2.4) | N.D. |
| 9 | N.D. | N.D. | N.D. | N.D. | N.D. | N.D. | N.D. |
| 10 | N.D. | N.D. | N.D. | N.D. | N.D. | N.D. | N.D. |
| 11 | 7 017.76 (2.10) | N.D. | N.D. | N.D. | N.D. | N.D. | N.D. |
| 12 | N.D. | 515.23 (7.9) | 942.10 (1.7) | N.D. | 8.42 (1.4) | N.D. | 2.81 (0.82) |
| 13 | N.D. | N.D. | 396.69 (6.3) | N.D. | 4.41 (9.8) | N.D. | 5.51 (6.1) |
| 14 | N.D. | N.D. | 731.82 (4.9) | N.D. | 17.83 (4.9) | N.D. | N.D. |
| 15 | N.D. | N.D. | N.D. | 2.59 (4.2) | 59.25 (2.0) | 4.01 (1.6) | 20.14 (3.9) |
| 16 | N.D. | N.D. | N.D. | N.D. | N.D. | N.D. | N.D. |
| 17 | 2 081.52 (3.2) | N.D. | N.D. | N.D. | N.D. | N.D. | N.D. |
| 18 | N.D. | N.D. | N.D. | N.D. | N.D. | N.D. | N.D. |
| 19 | N.D. | N.D. | N.D. | N.D. | N.D. | N.D. | N.D. |
| 20 | 1 114.48 (6.8) | N.D. | N.D. | N.D. | 6.63 (1.1) | N.D. | N.D. |
| 21 | N.D. | N.D. | N.D. | N.D. | N.D. | N.D. | N.D. |
| 22 | 43 599.96 (1.8) | N.D. | N.D. | 2.58 (1.6) | 10.66 (0.26) | N.D. | N.D. |
| 23 | N.D. | N.D. | N.D. | N.D. | 9.24 (28) | N.D. | N.D. |
| 24 | 26 726.40 (9.8) | N.D. | 49.46 (4.8) | N.D. | 15.10 (7.5) | N.D. | N.D. |

N.D. Not Detected

## S8. Chromatogram and MS^2^ spectrum- samples and references

### **S8.1** Suspect screening


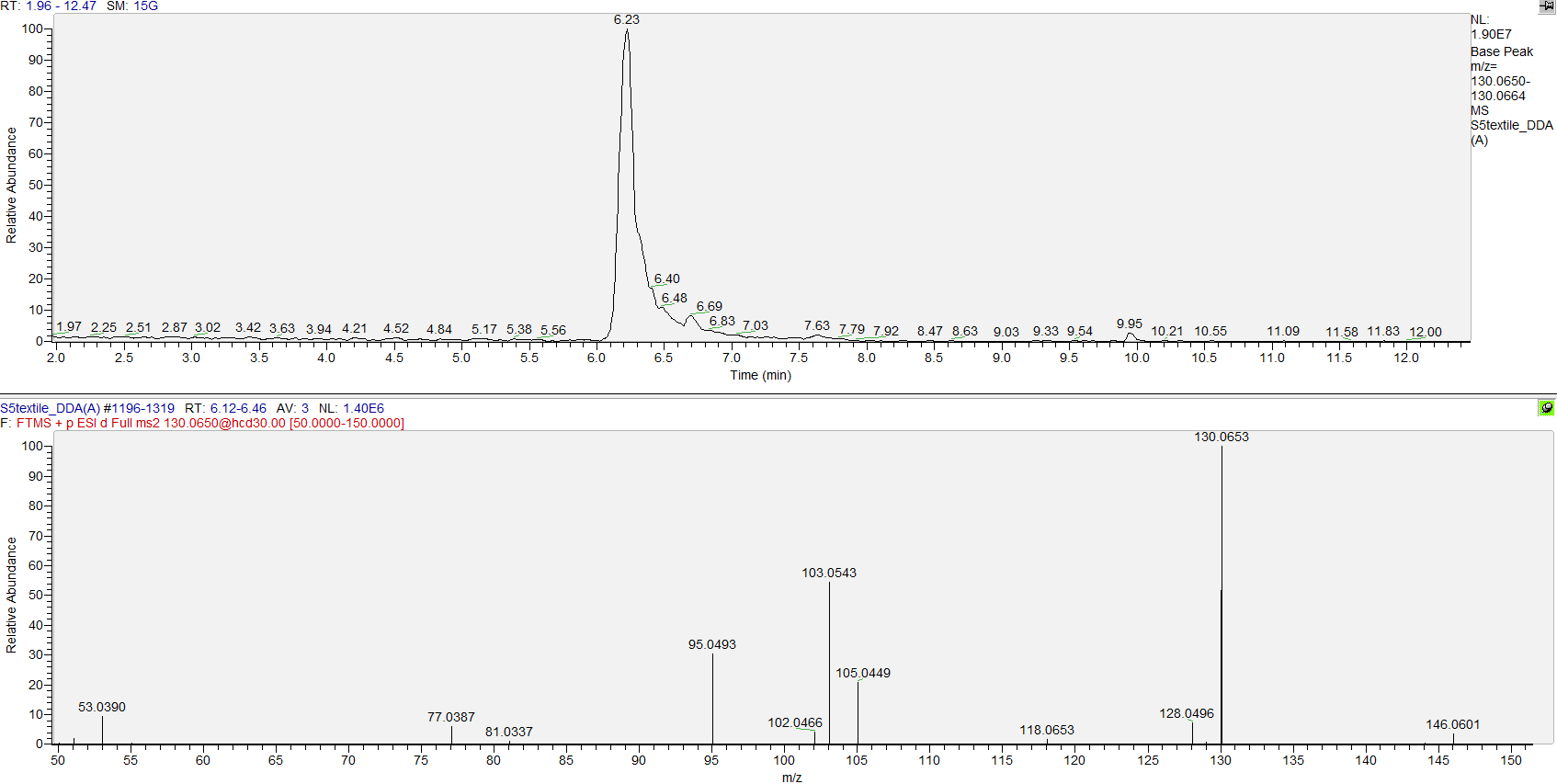


**Figure S8.1:1.** Quinoline chromatogram and MS^2^ spectrum in sample 5.


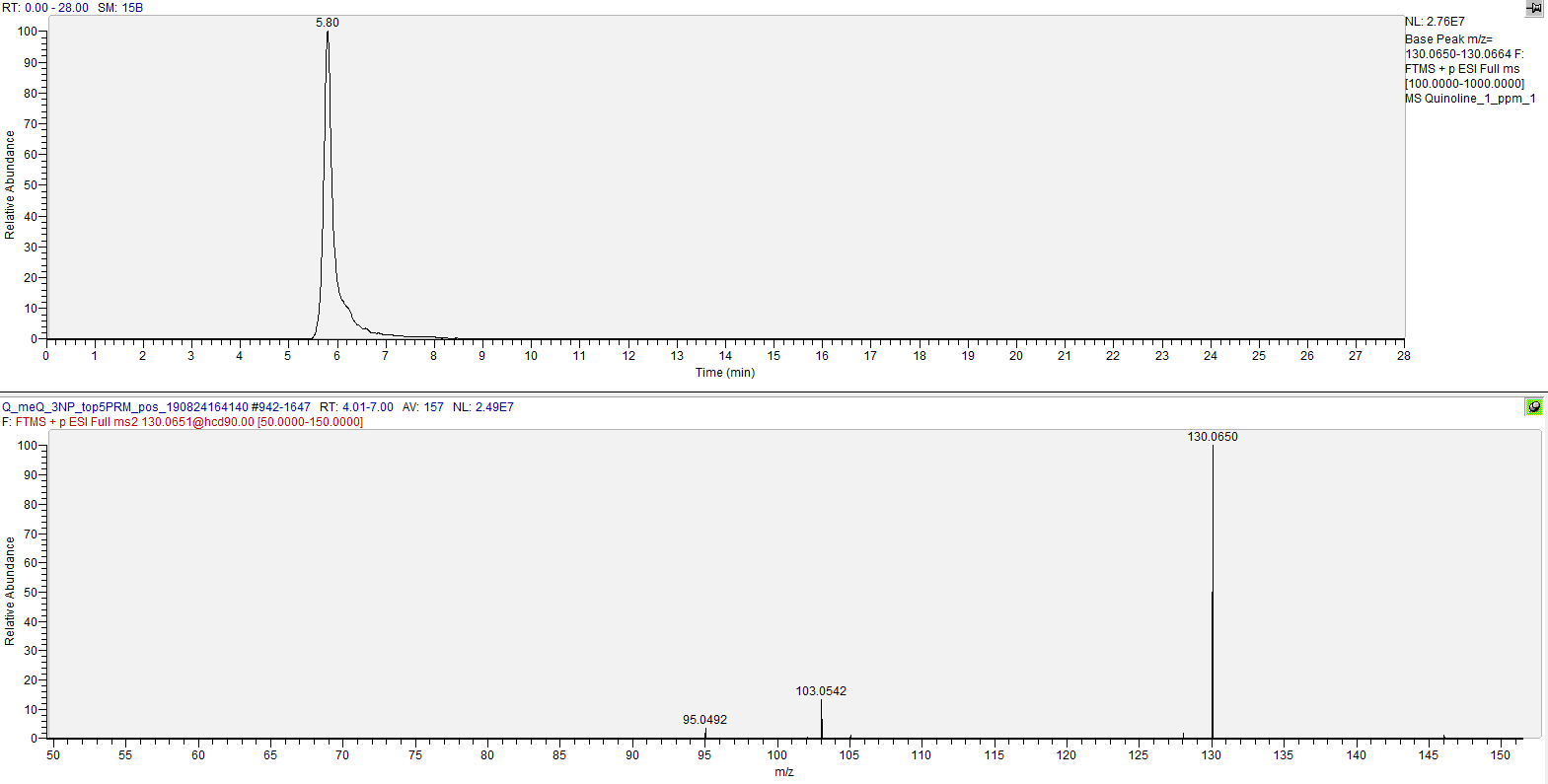


**Figure S8.1:2.** Reference standard chromatogram and MS^2^ spectrum of quinoline.


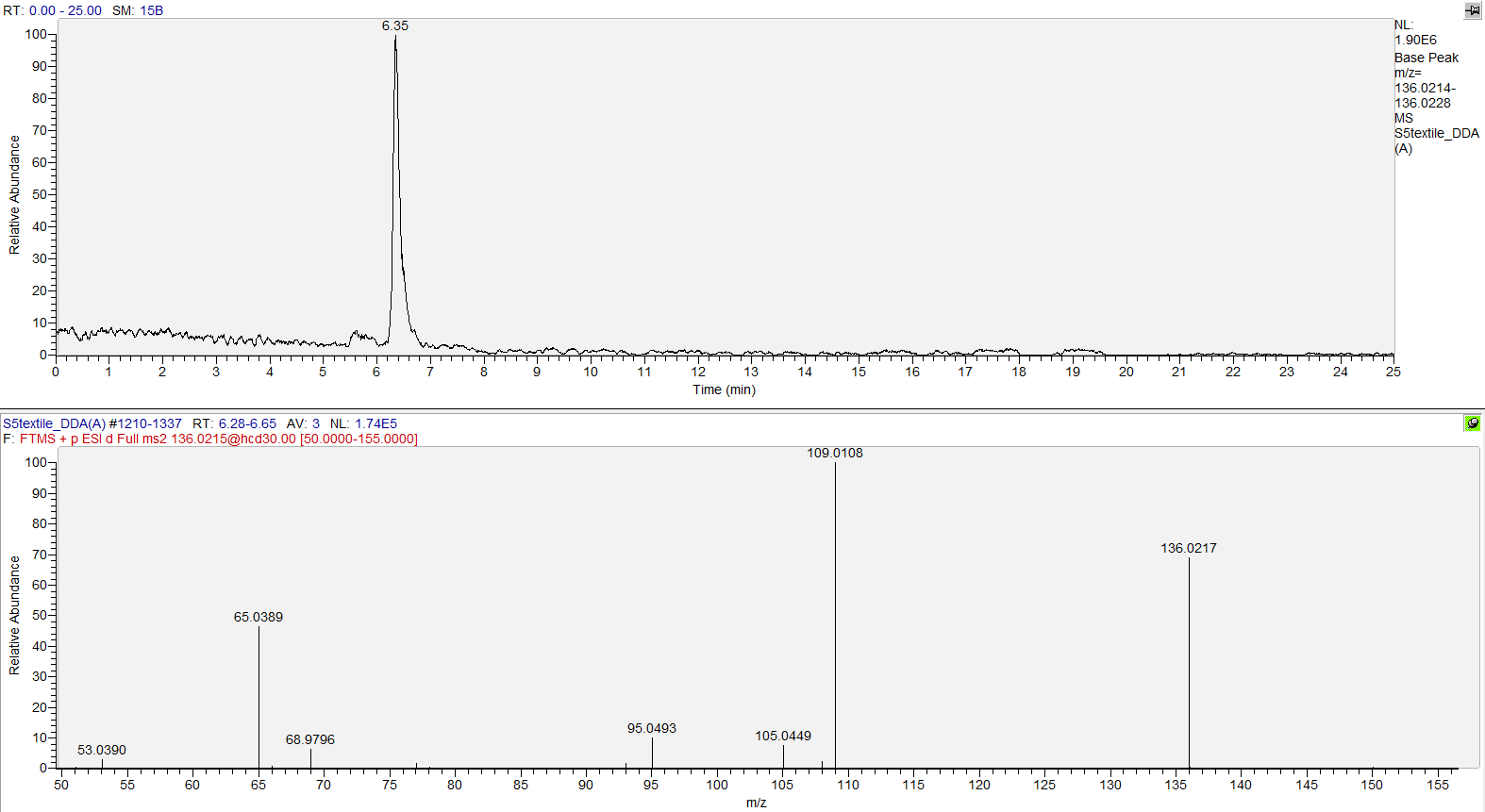


**Figure S8.1:3.** Benzothiazole (BT) chromatogram and MS^2^ spectrum in sample 5.


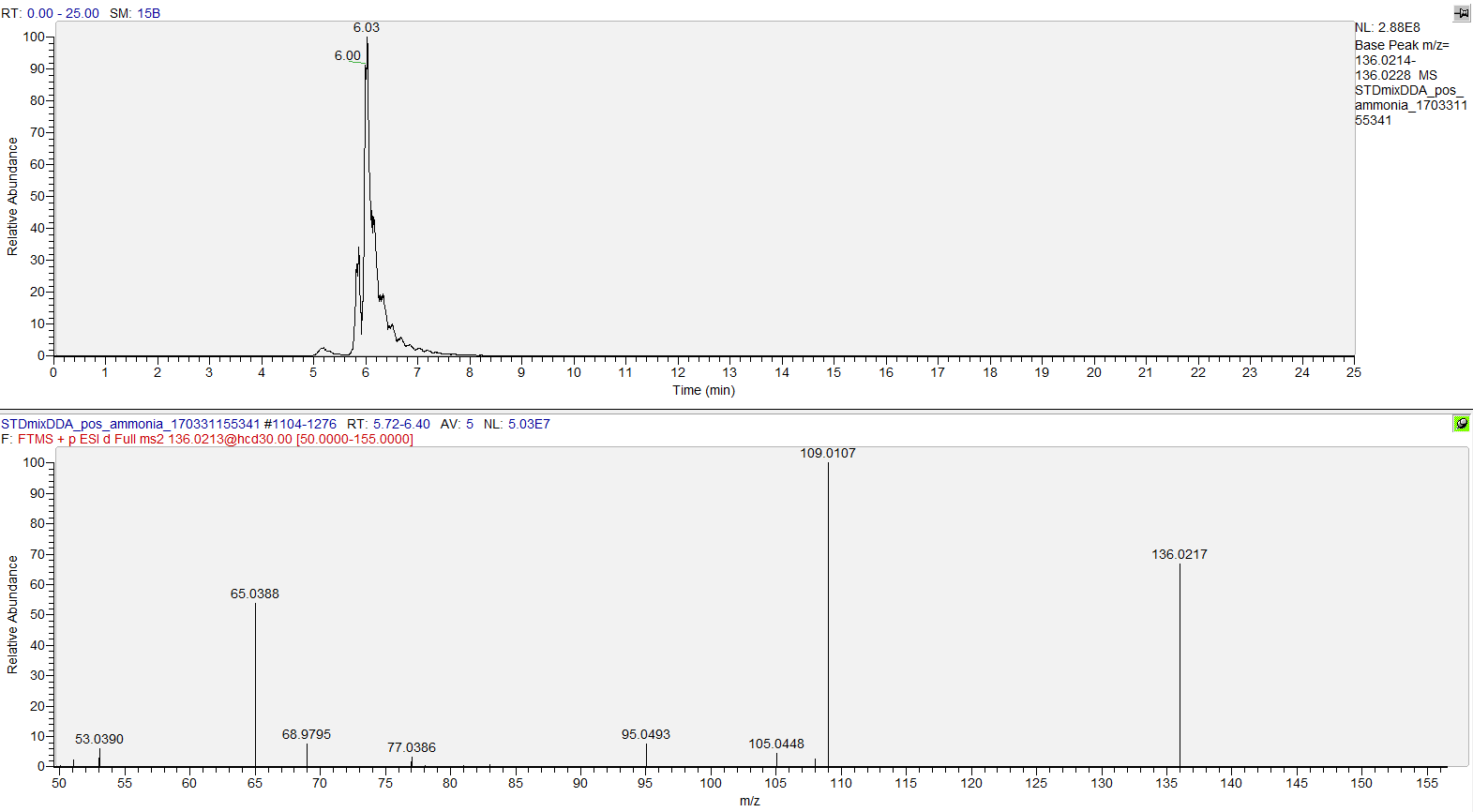


**Figure S8.1:4.** Reference standard chromatogram and MS^2^ spectrum of benzothiazole (BT).


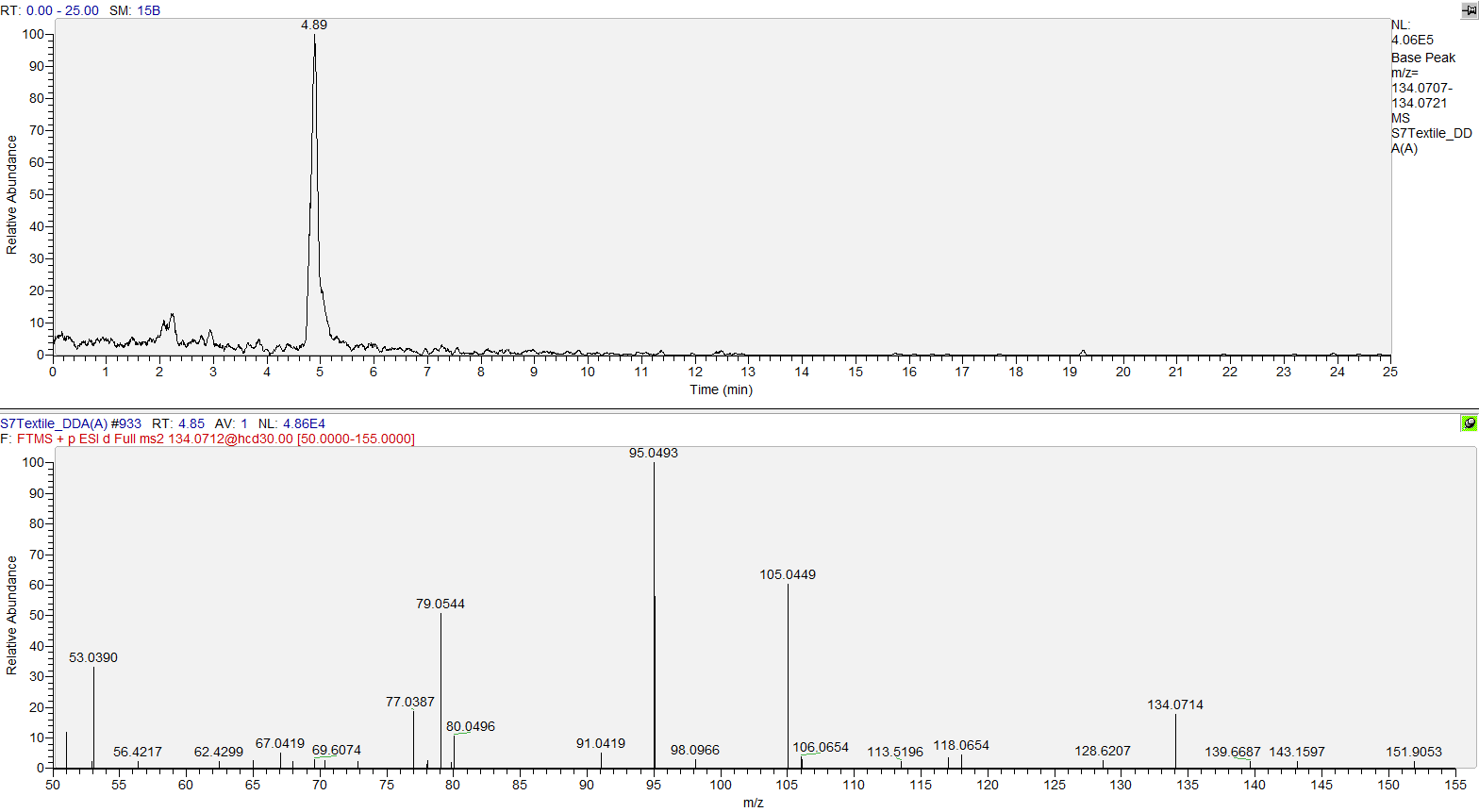


**Figure S8.1:5.** Tolyltriazole (TTri) chromatogram and MS^2^ in sample 9.


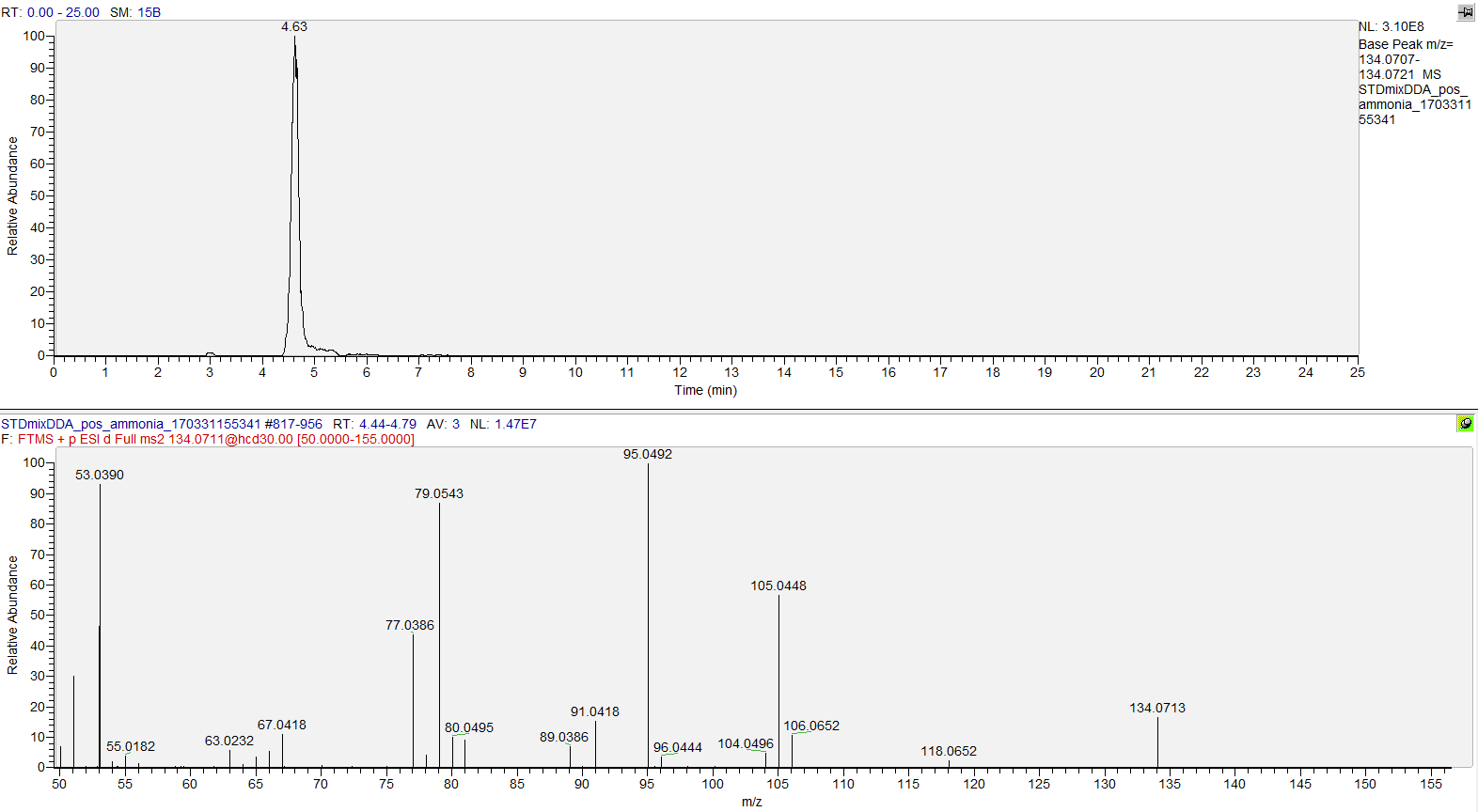


**Figure S8.1:6.** Reference standard chromatogram and MS^2^ spectrum of 5-methylbenzotriazole (5-TTri).


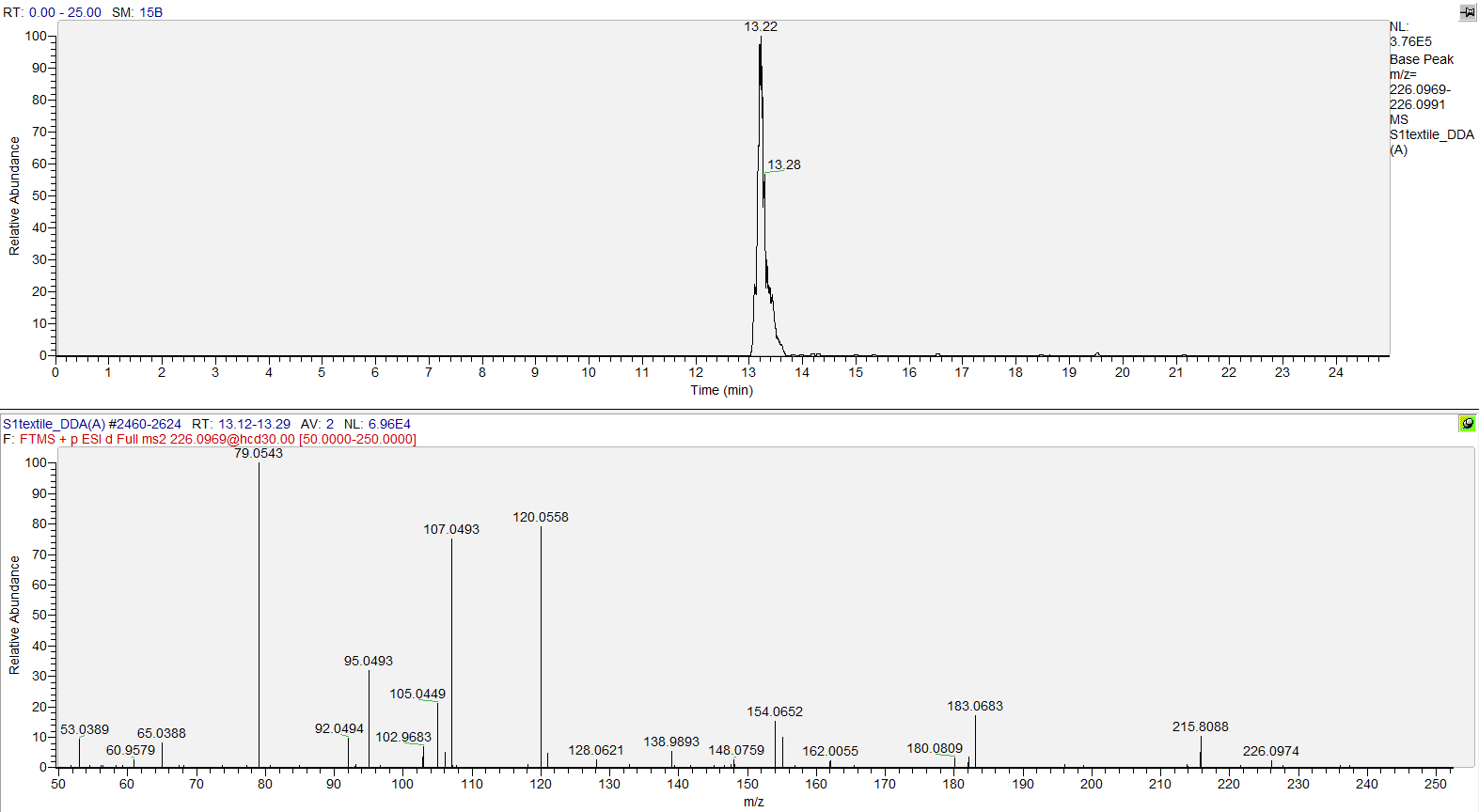


**Figure S8.1:7.** Chromatogram and MS^2^ spectrum of 2-(benzotriazol-2-yl)-4-methylphenol (UV-P) in sample 1.


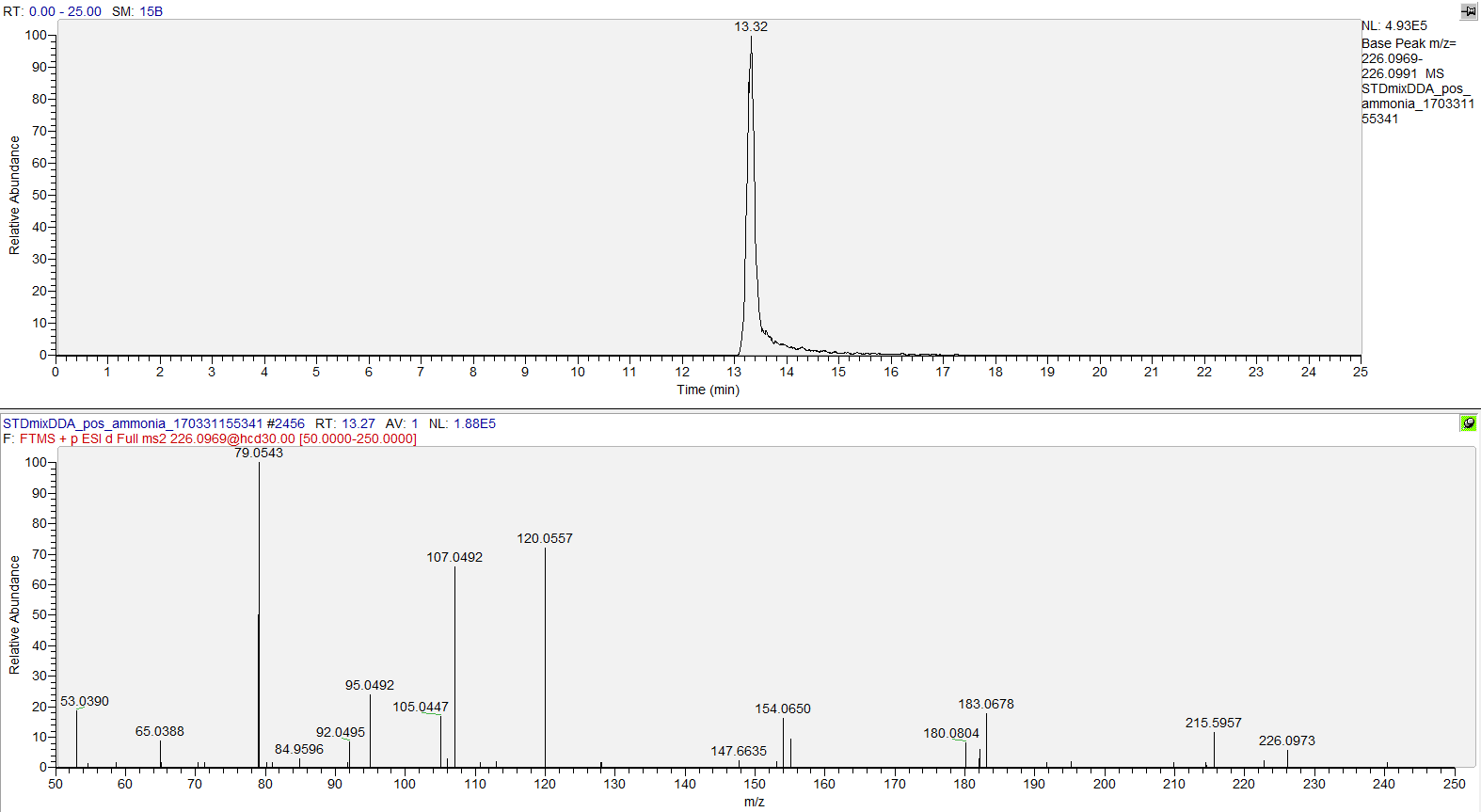


**Figure S8.1:8.** Reference standard chromatogram and MS^2^ spectrum of 2-(benzotriazol-2-yl)-4-methylphenol (UV-P).


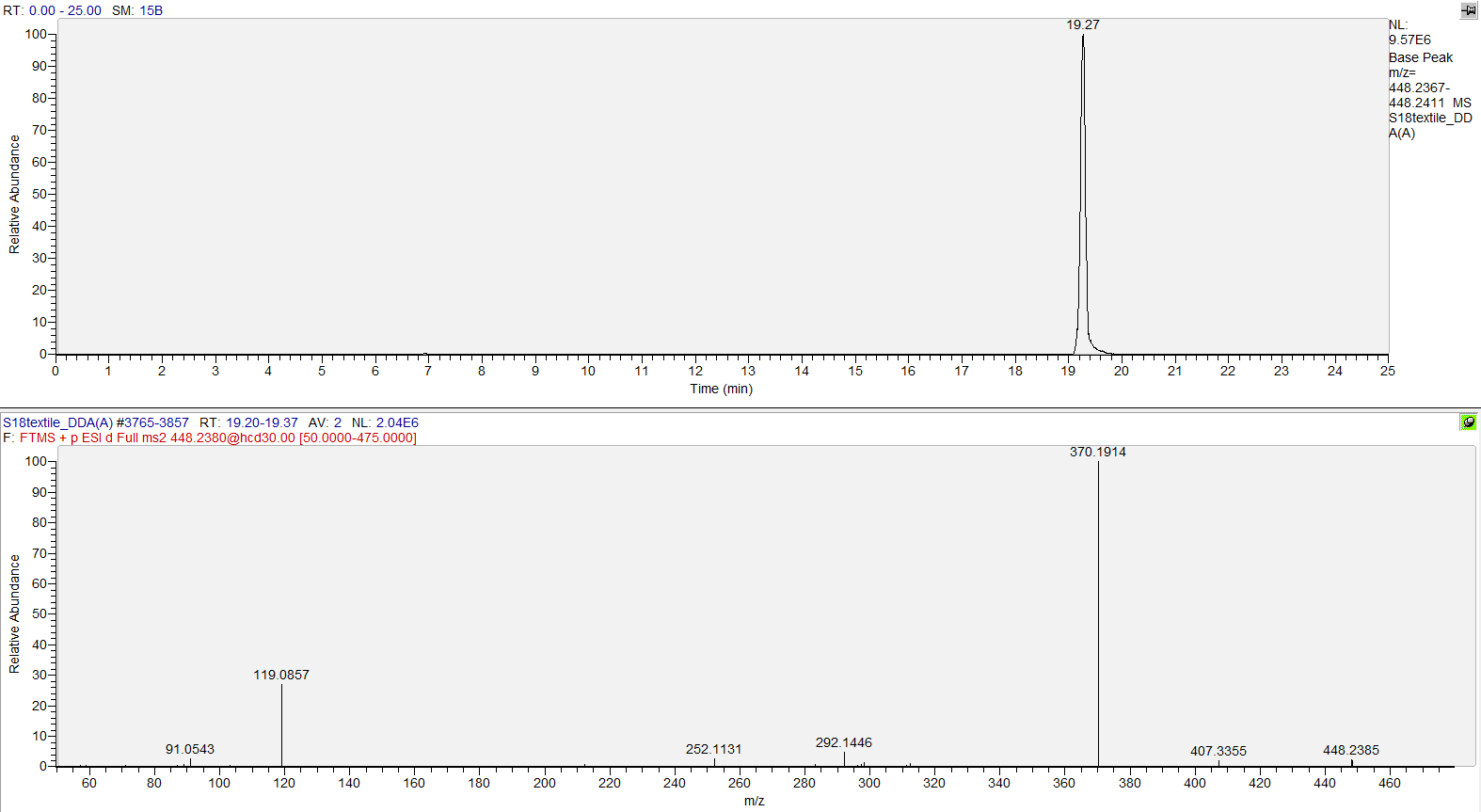


**Figure S8.1:9.** Chromatogram and MS^2^ spectrum of 2-(2H-benzotriazol-2-yl)-4,6-bis(1-methyl-1-phenylethyl) phenol (UV-234) in sample 18.


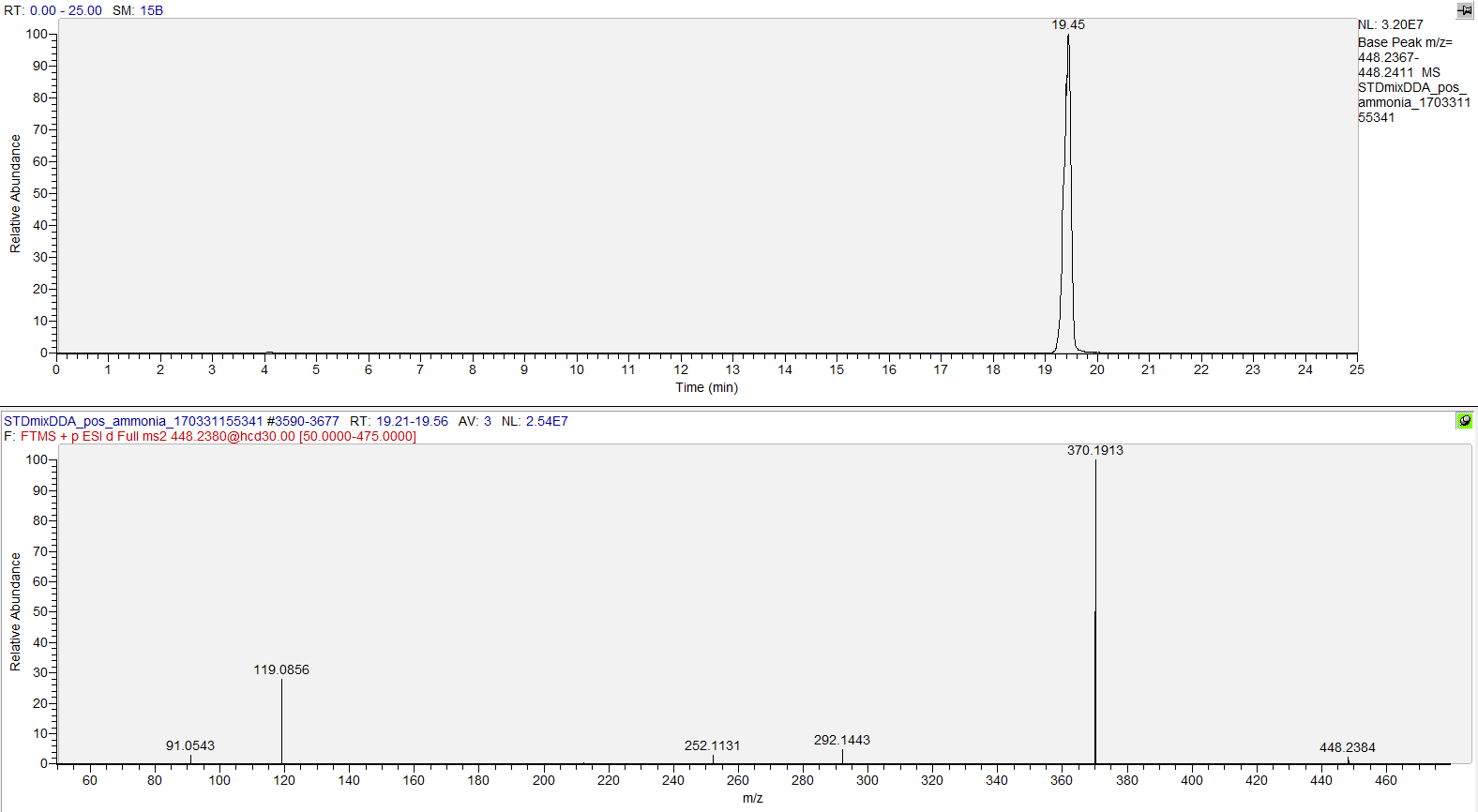


**Figure S8.1:10.** Reference standard chromatogram and MS^2^ spectrum of 2-(2H-benzotriazol-2-yl)-4,6-bis(1-methyl-1-phenylethyl) phenol (UV-234).


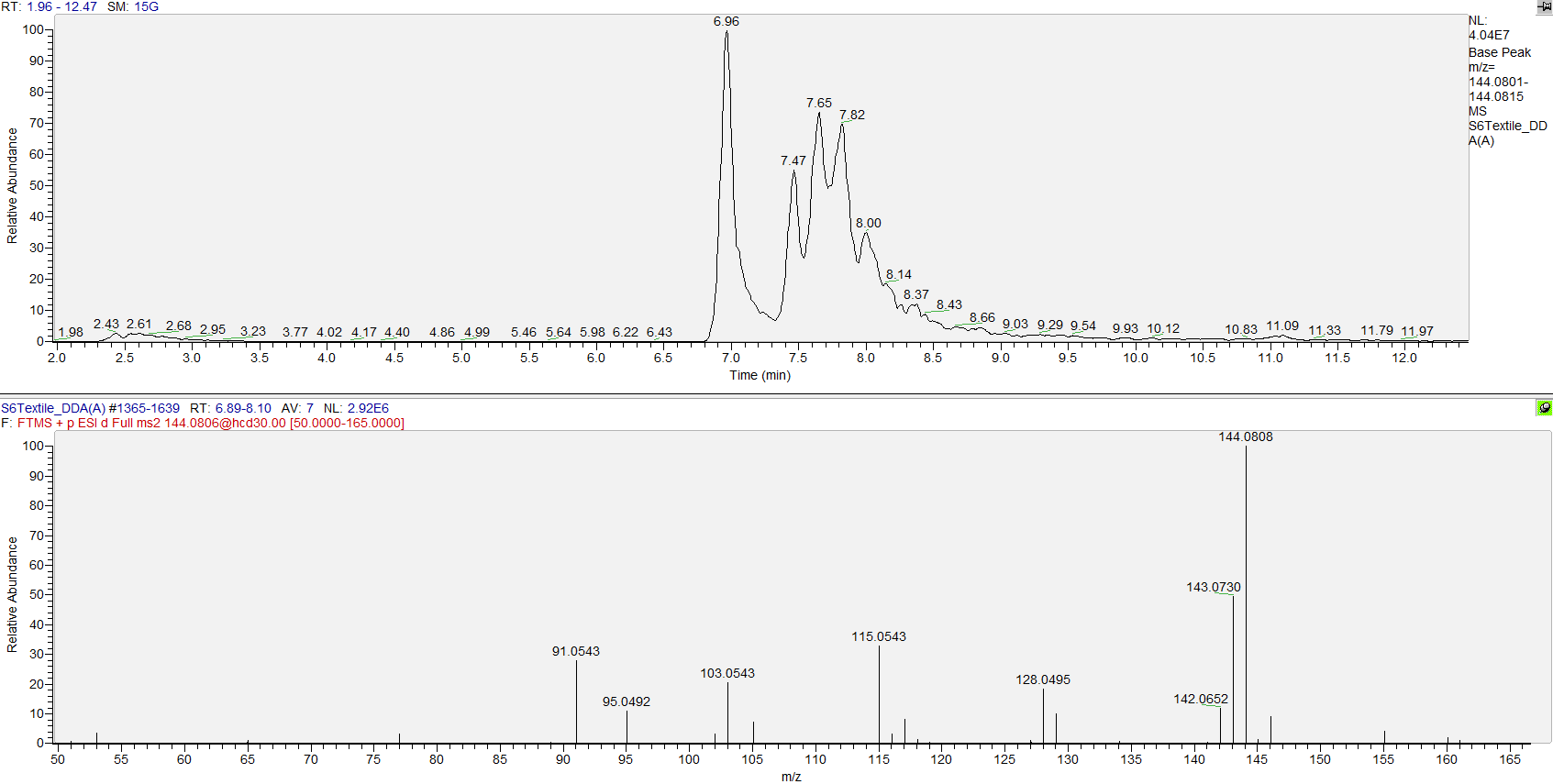
**Figure S8.1:11.** Chromatogram and MS^2^ spectrum of methylquinoline in sample 6.


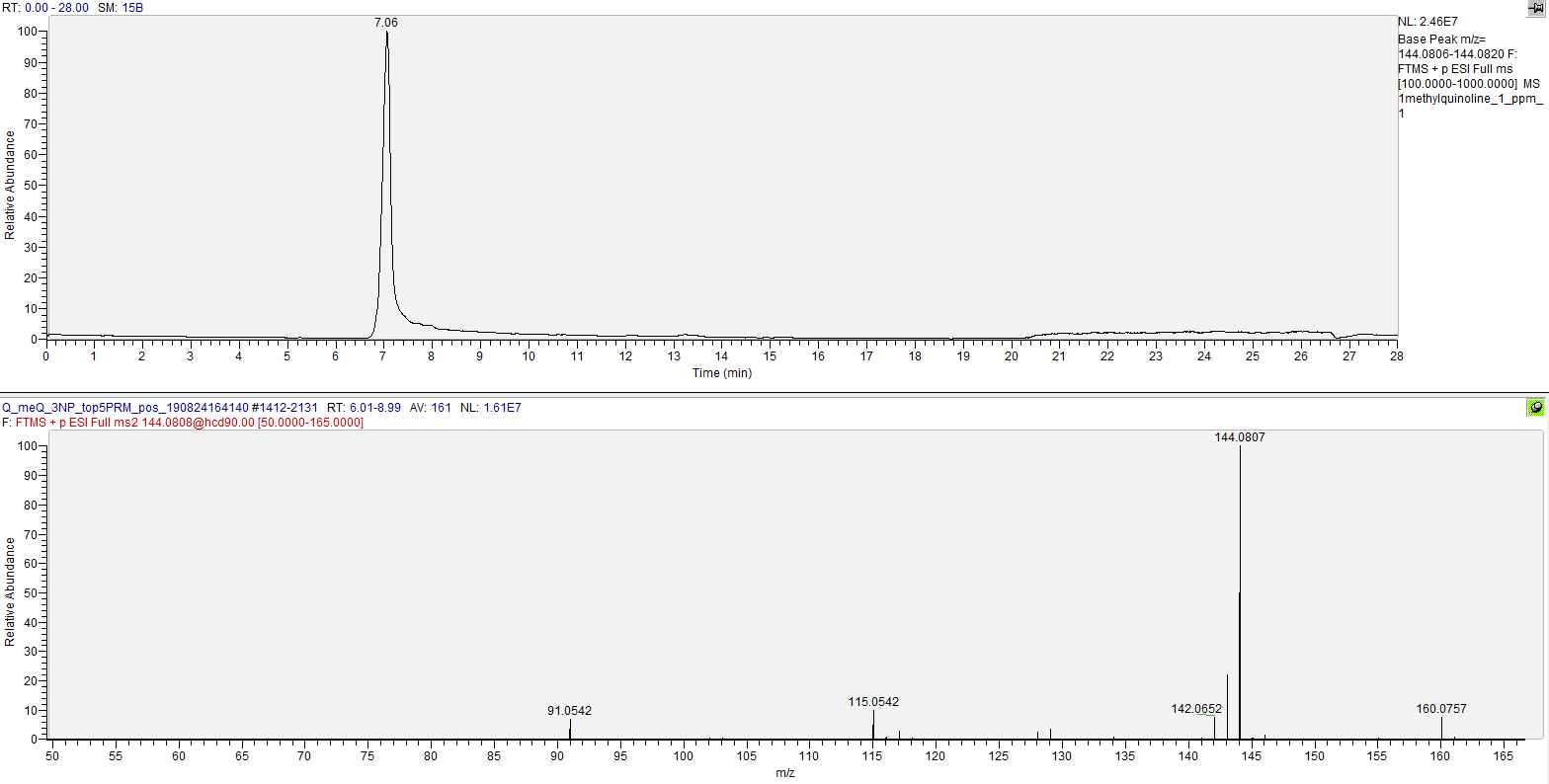


**Figure S8.1:12.** Reference standard chromatogram and MS^2^ spectrum of 1-methylquinoline.


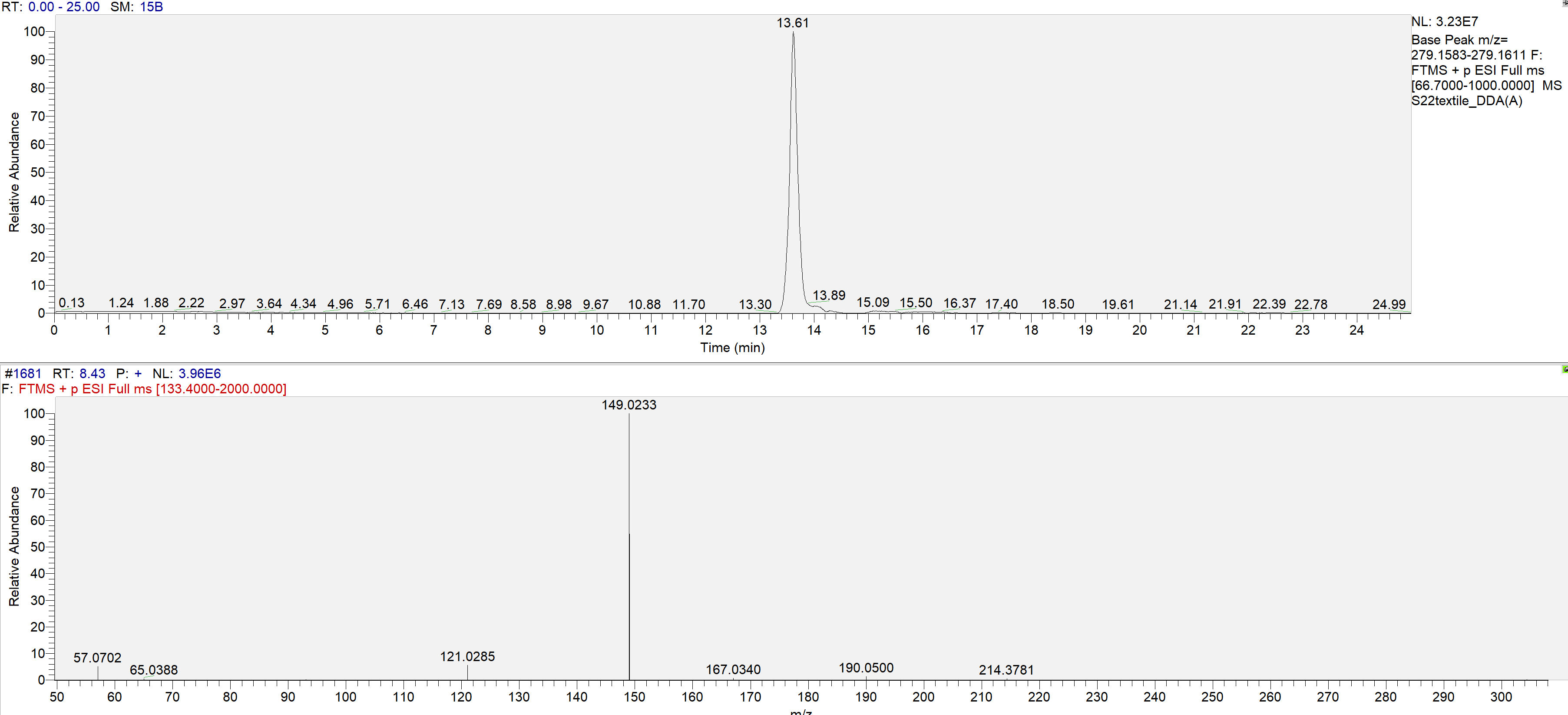


**Figure S8.1:13.** Chromatogram and MS^2^ spectrum of diisobutyl phthalate (DiBP) in sample 22.


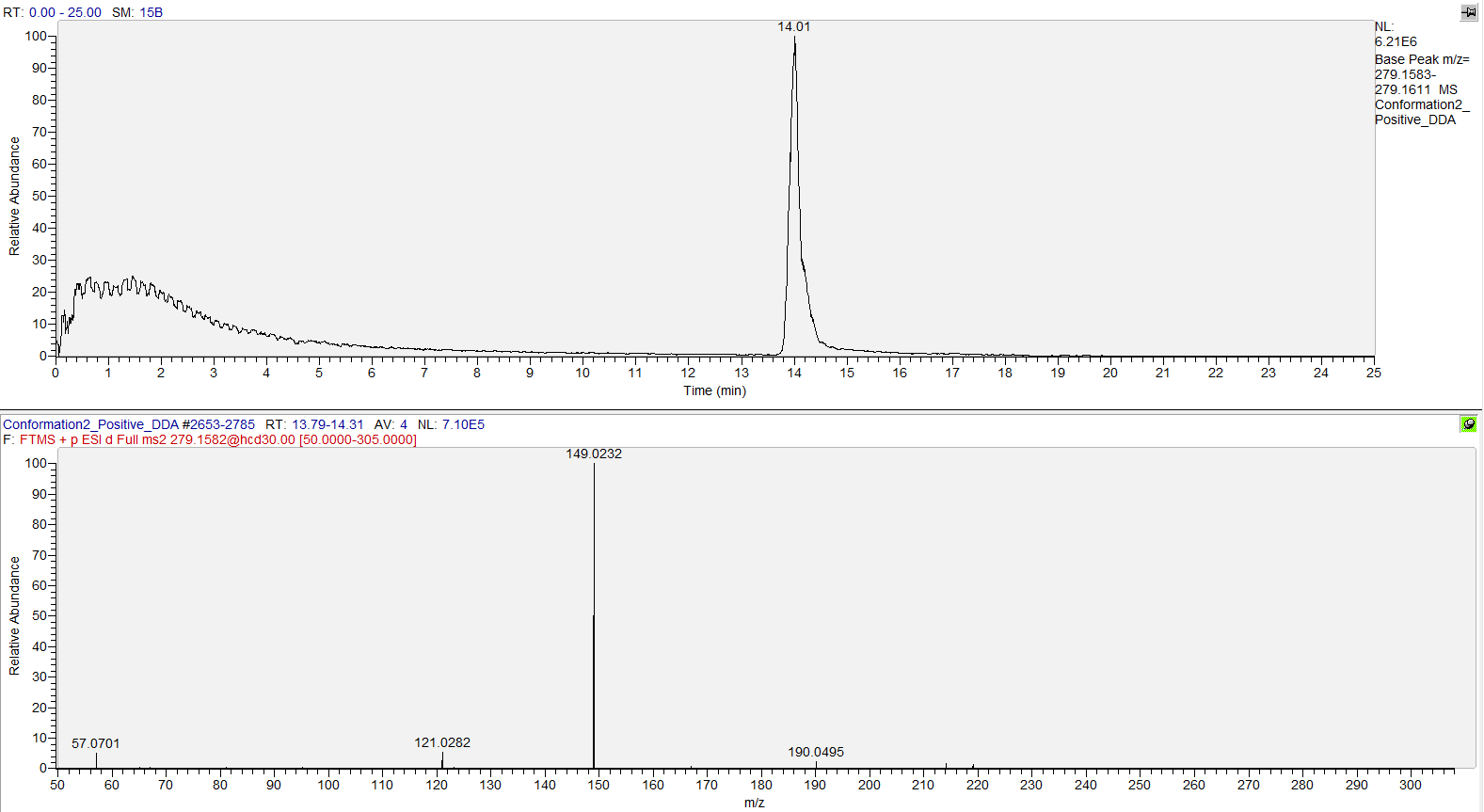


**Figure S8.1:14.** Reference standard chromatogram and MS^2^ spectrum of diisobutyl phthalate (DiBP).


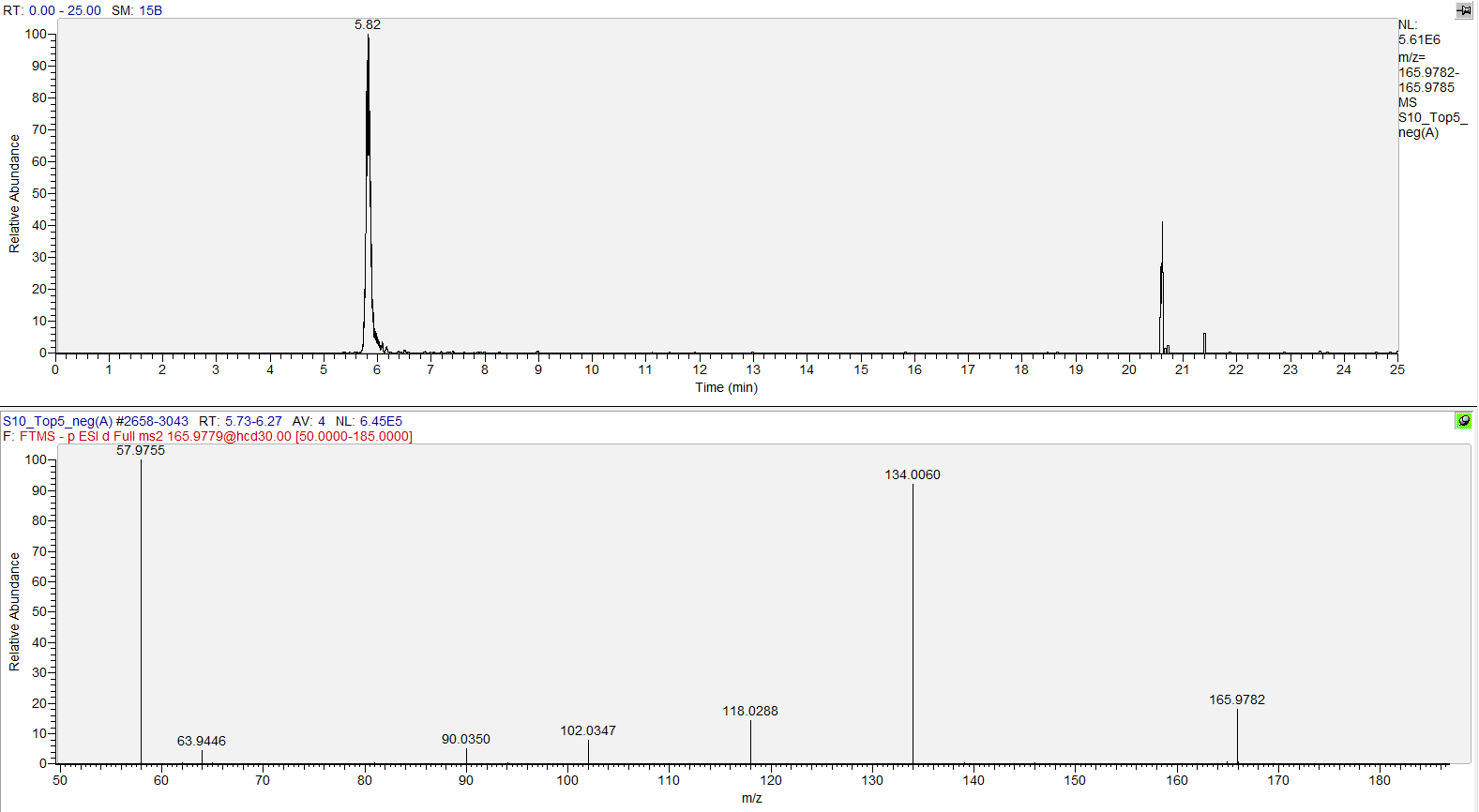


**Figure S8.1:15.** Chromatogram and MS^2^ spectrum of 2-mercaptobenzothiazole (MTB) in sample 10.


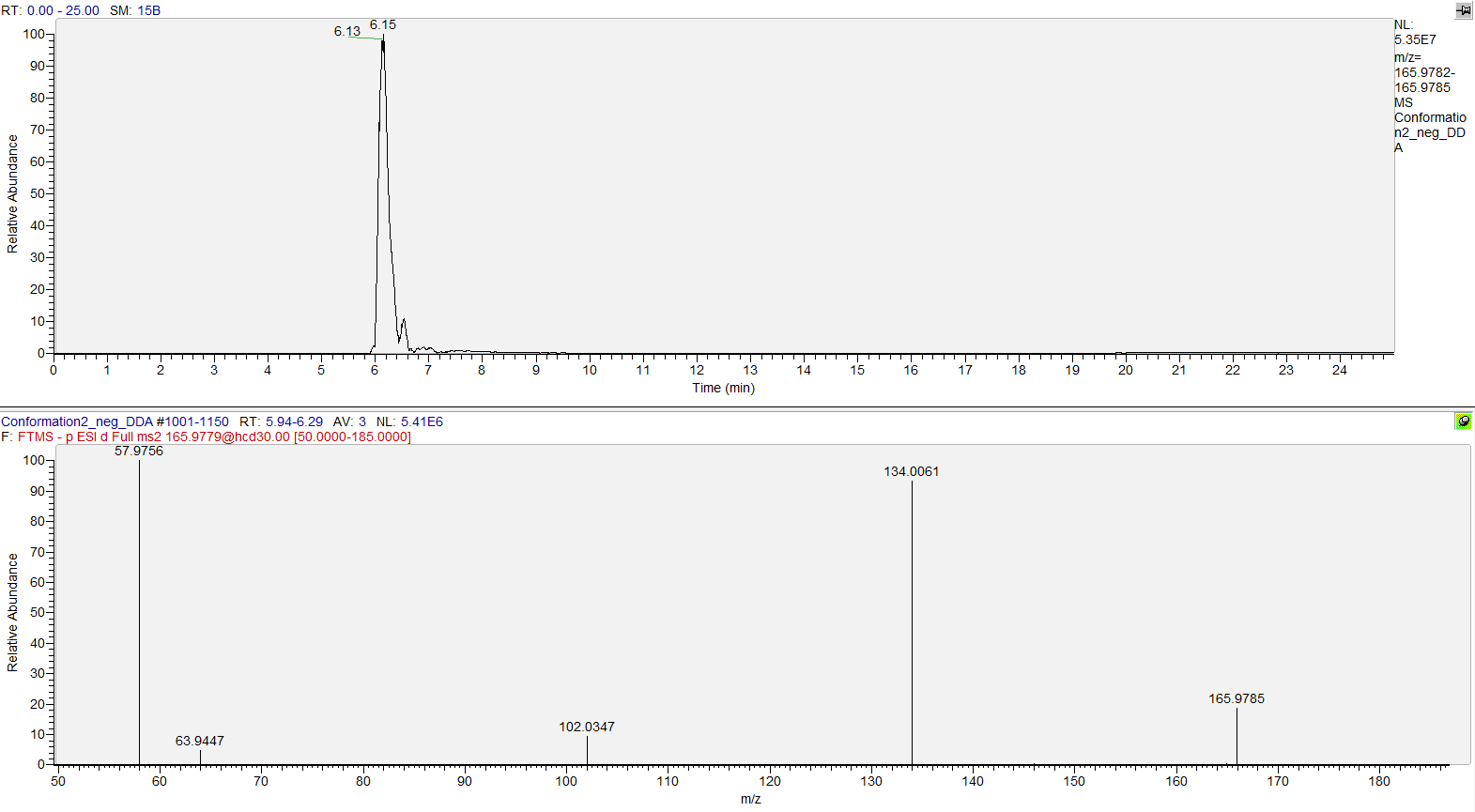


**Figure S8.1:16.** Reference standard chromatogram and MS^2^ spectrum of 2-mercaptobenzothiazole (MBT).


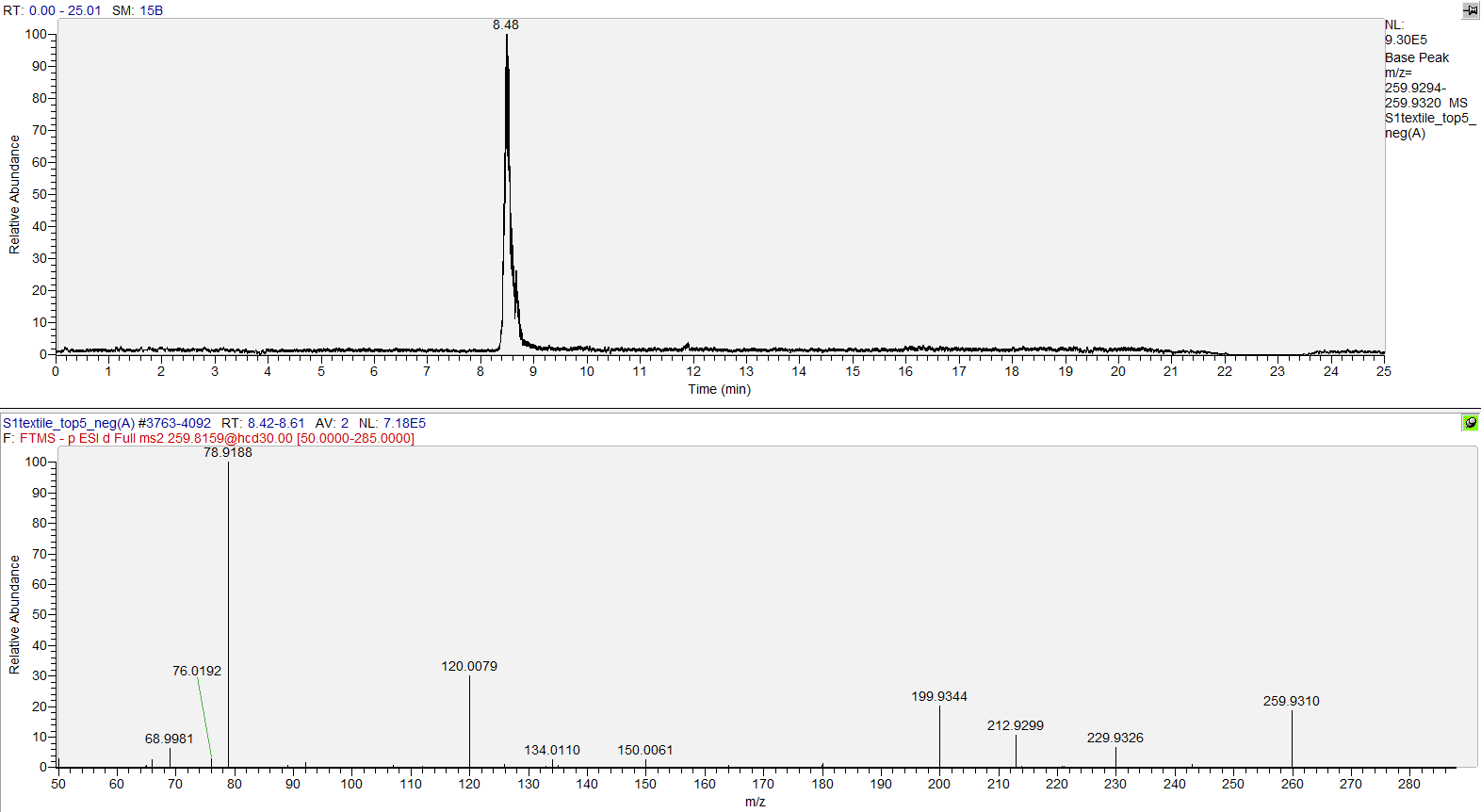


**Figure S8.1:17.** Chromatogram and MS^2^ spectrum of 2-bromo-4,6-dinitroaniline in sample.


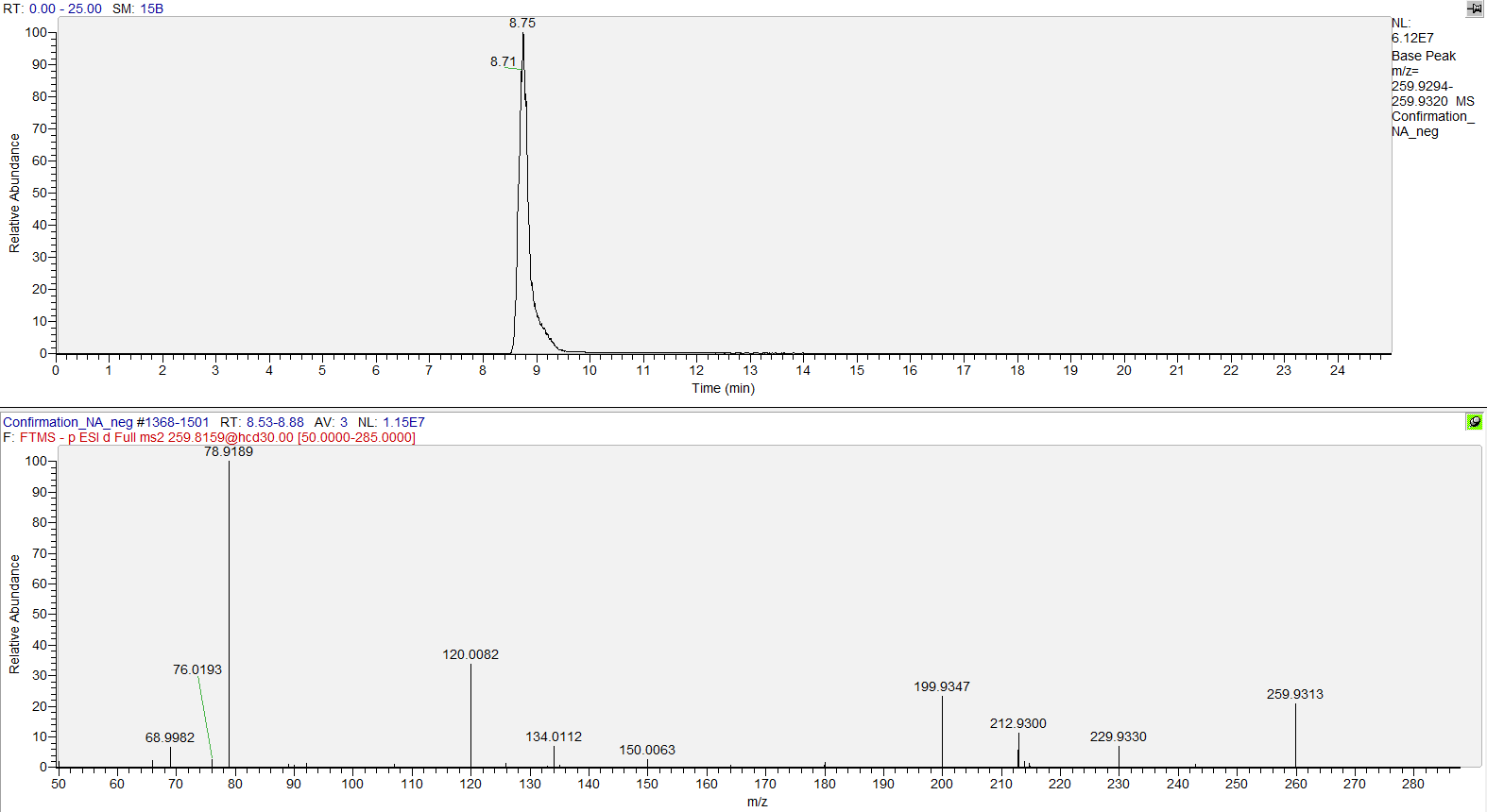


**Figure S8.1:18.** Reference standard chromatogram and MS^2^ spectrum of 2-bromo-4,6-dinitroaniline.


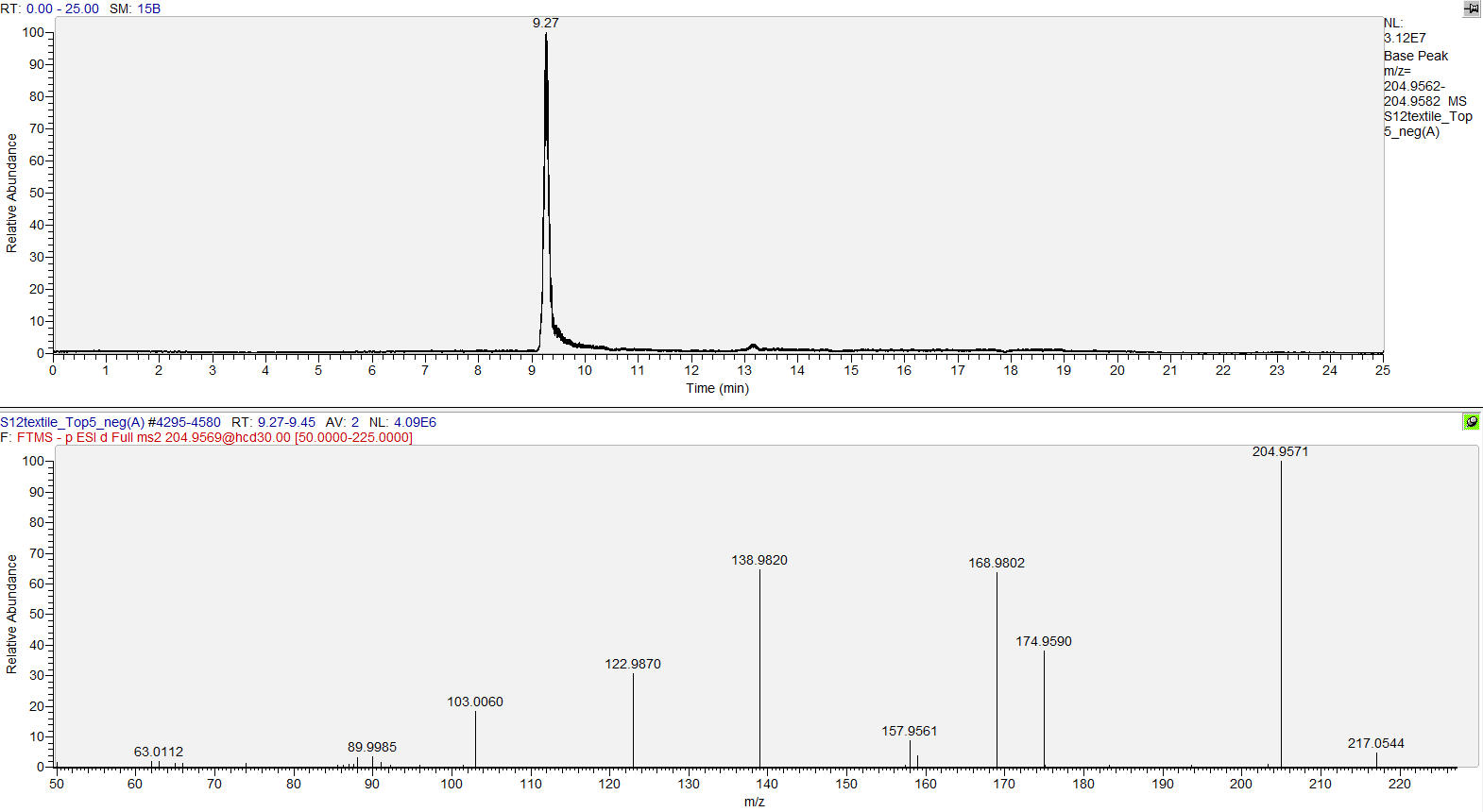


**Figure S8.1:19.** Chromatogram and MS^2^ spectrum of 2,6-dichloro-4-nitroaniline in sample 12.


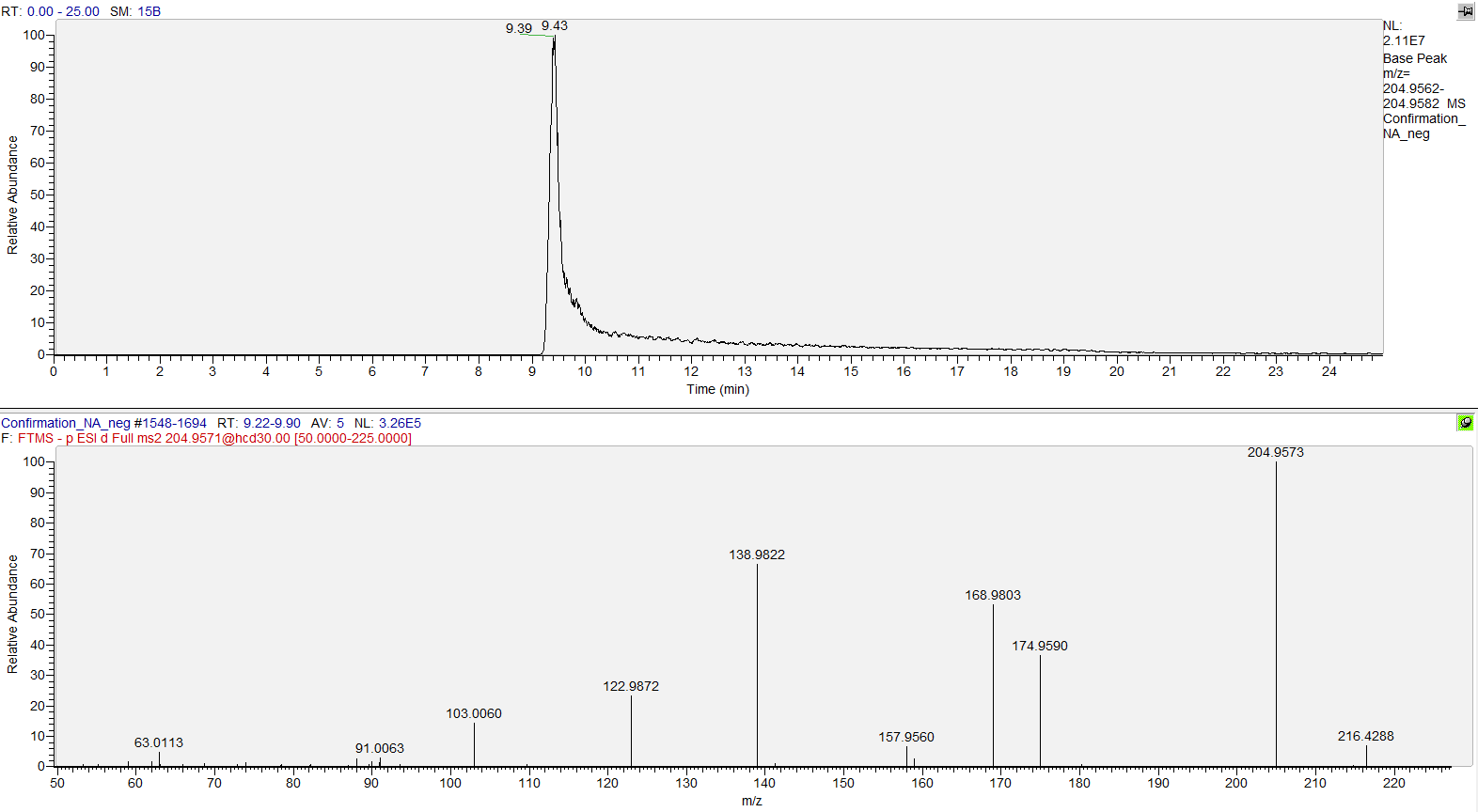


**Figure S8.1:20.** Reference standard chromatogram and MS^2^ spectrum of 2,6-dichloro-4-nitroaniline.


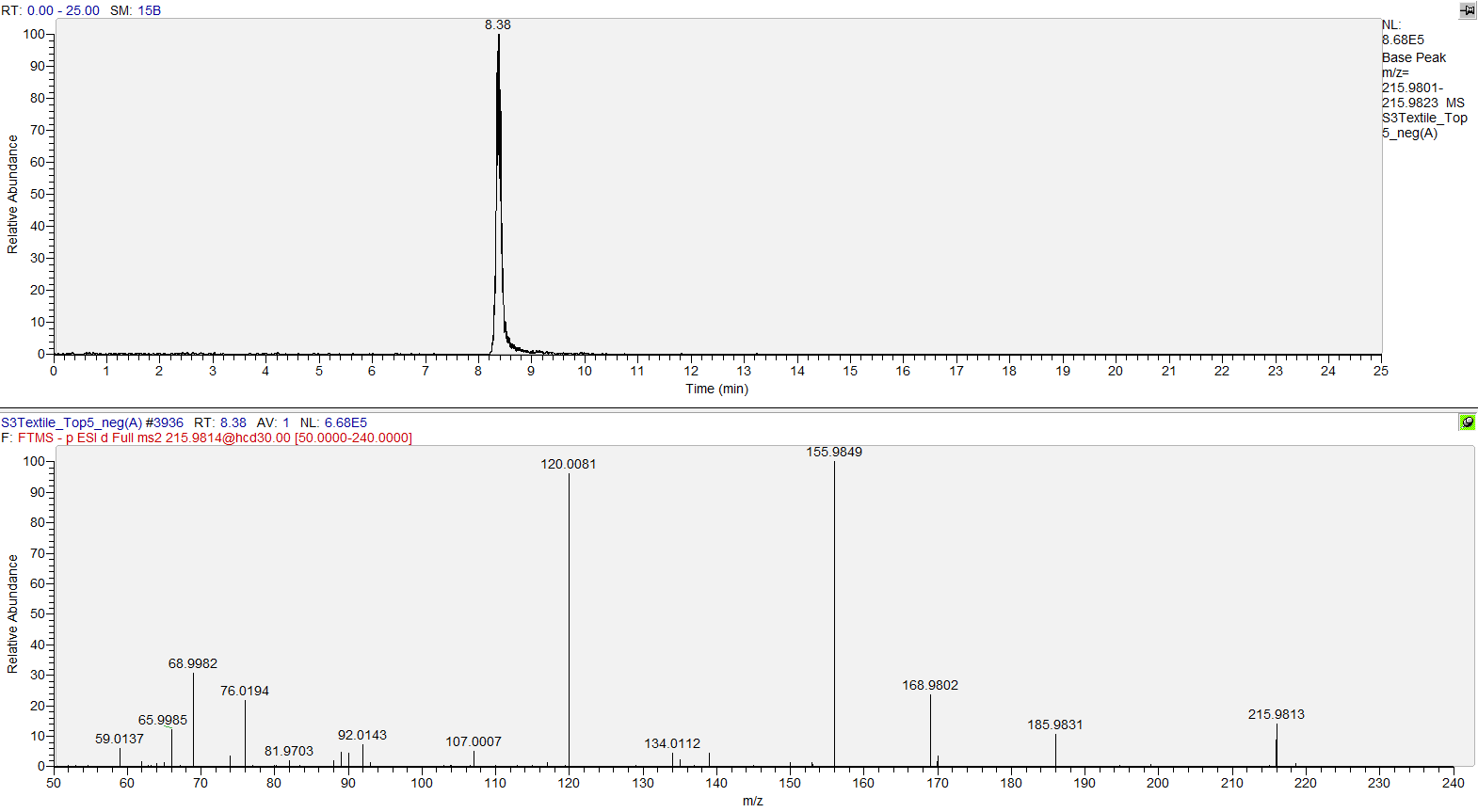


**Figure S8.1:21.** Chromatogram and MS^2^ spectrum of 6-chloro-2,4-dinitroaniline in sample 3.


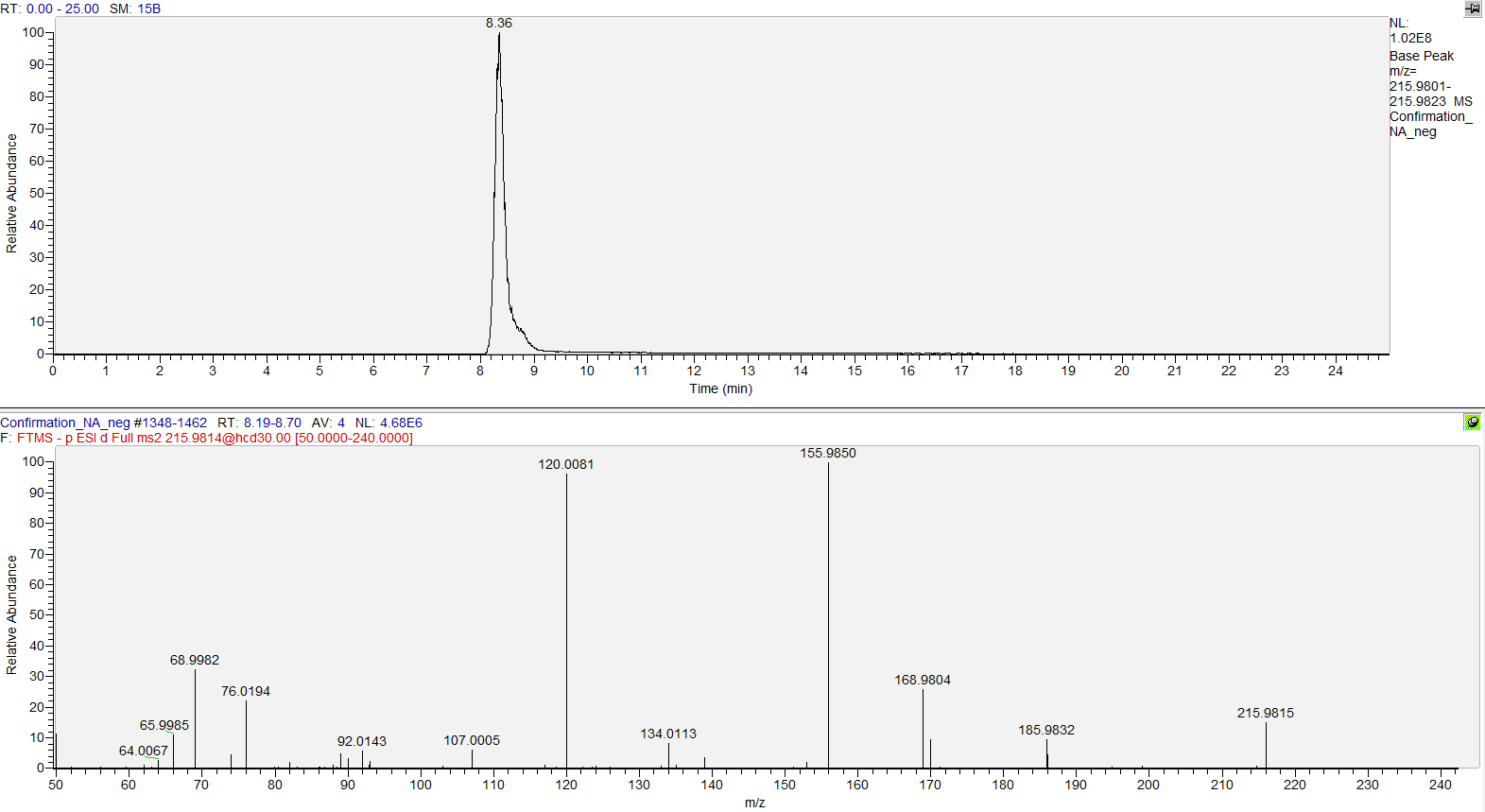


**Figure S8.1:22.** Reference standard chromatogram and MS^2^ spectrum of 6-chloro-2,4-dinitroaniline.


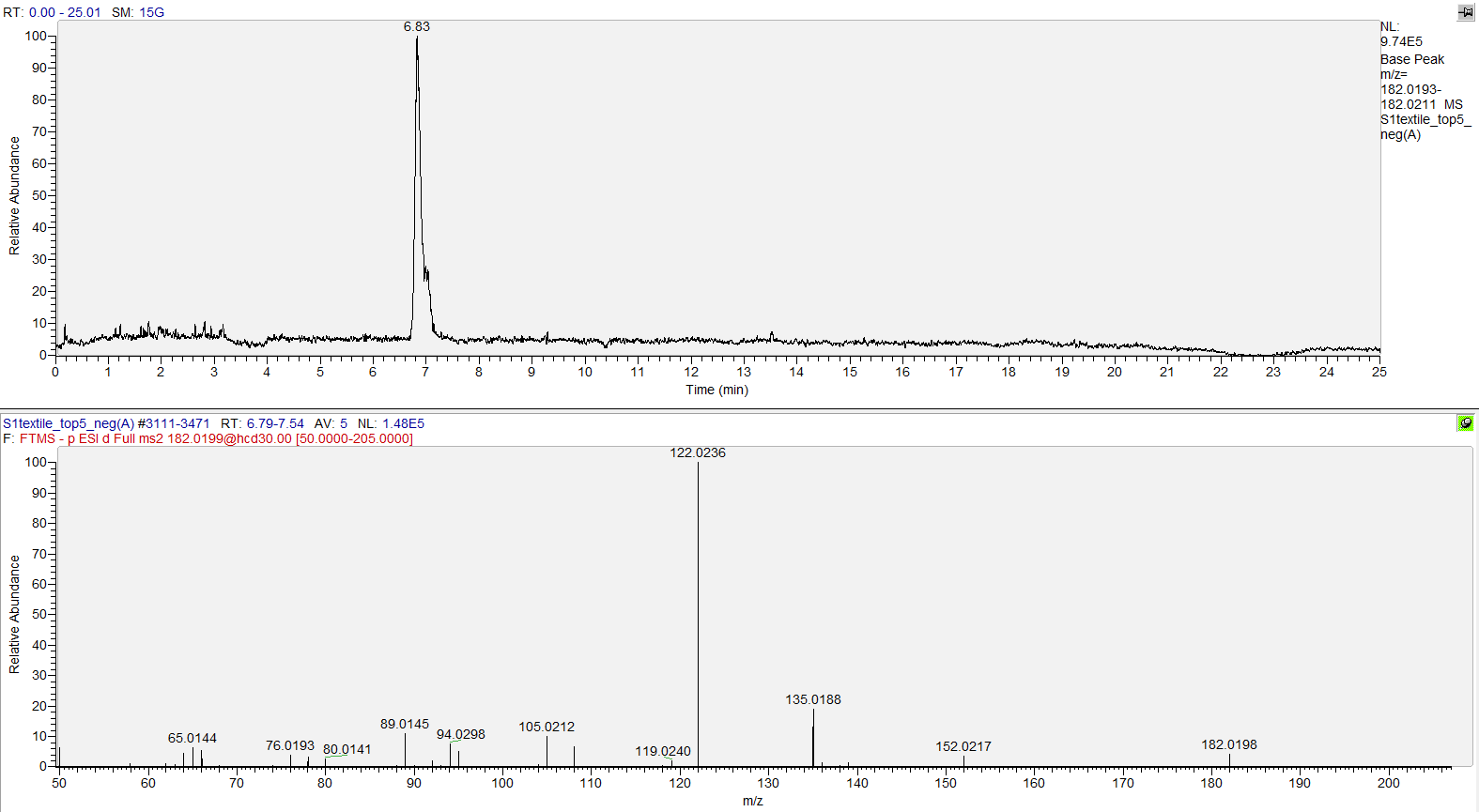


**Figure S8.1:23.** Chromatogram and MS^2^ spectrum of 2,4-dinitroaniline in sample 1


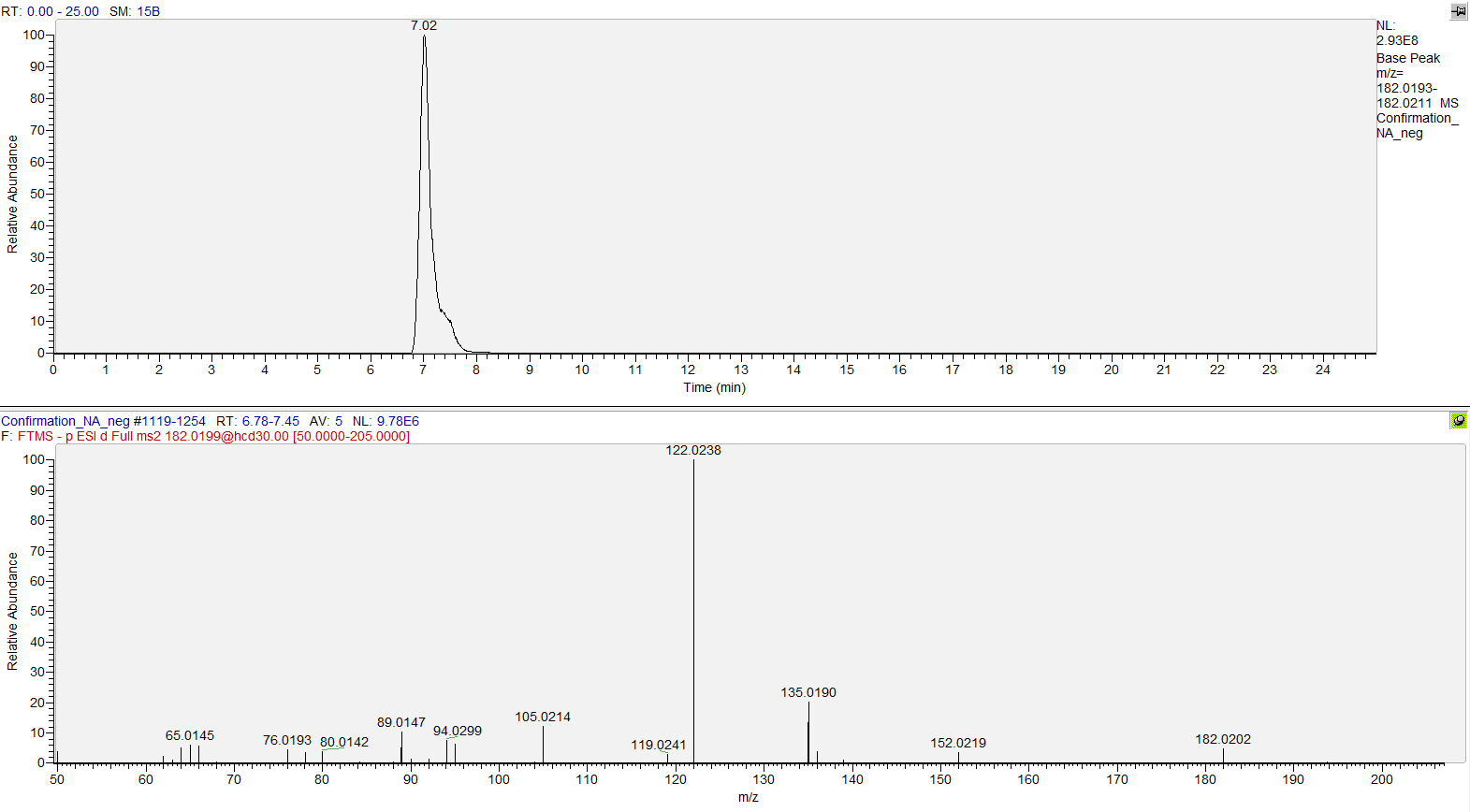


**Figure S8.1:24.** Reference standard chromatogram and MS^2^ spectrum of 2,4-dinitroaniline.


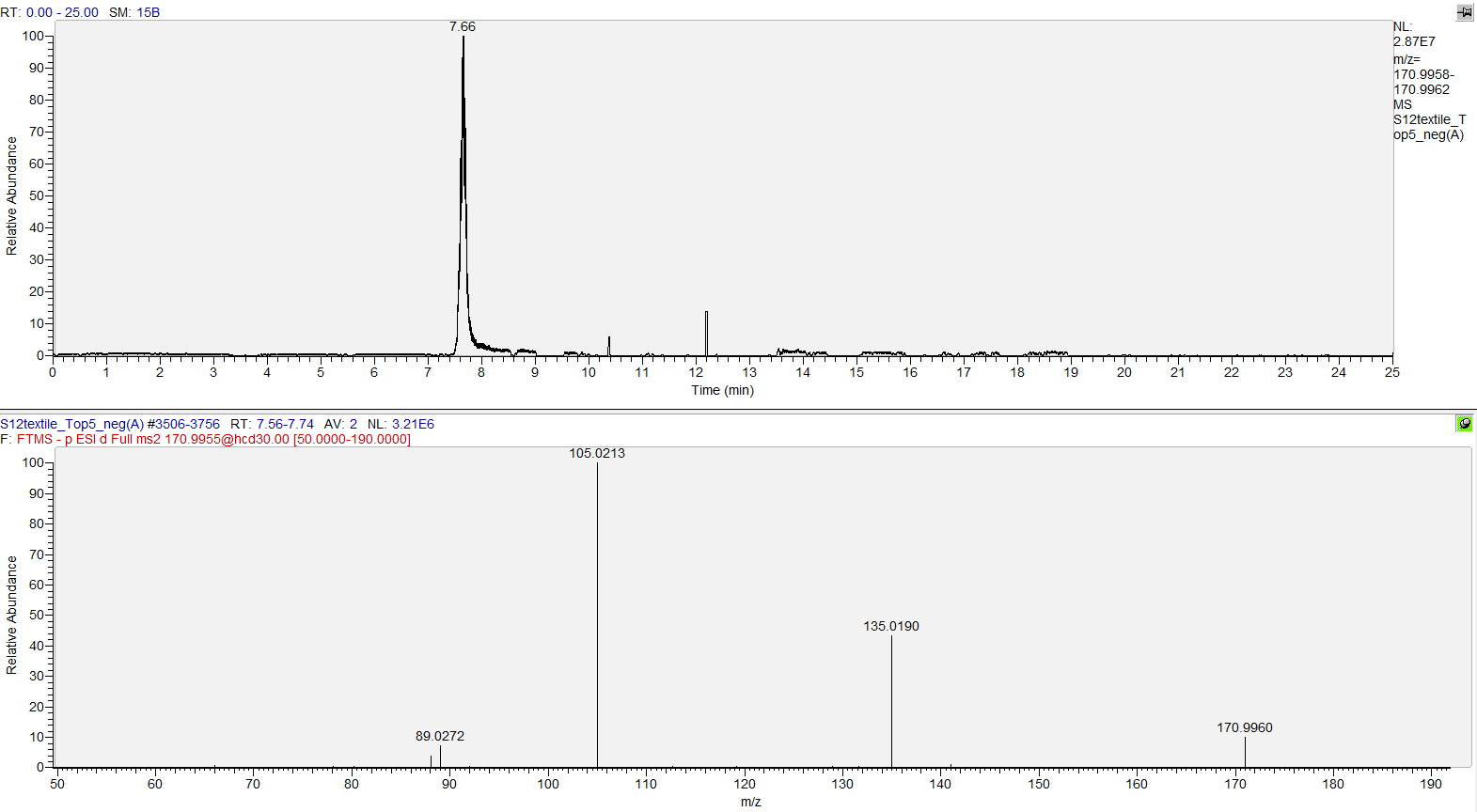


**Figure S8.1:25.** Chromatogram and MS^2^ spectrum of 2-chloro-4-nitroaniline in sample 12.


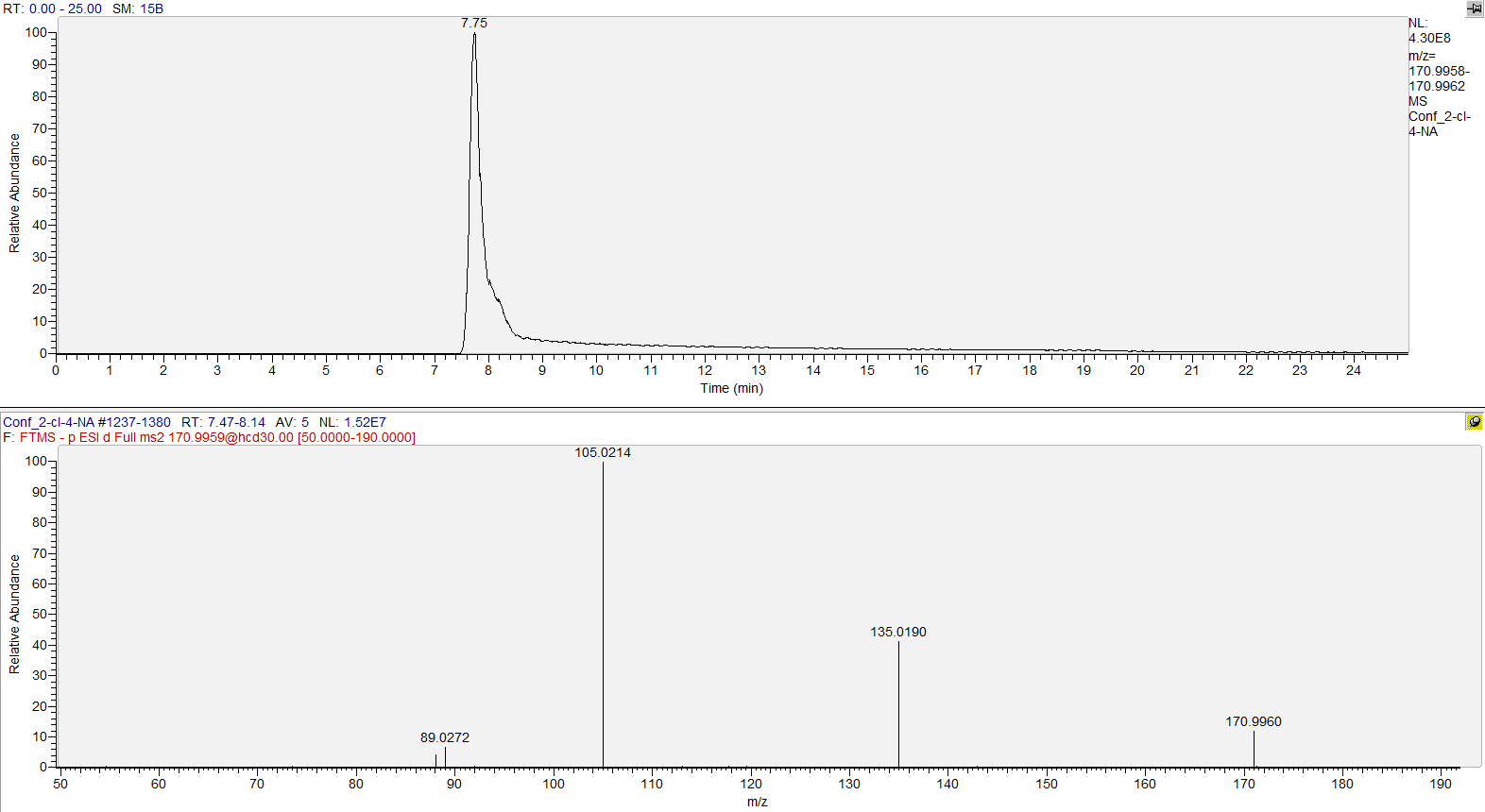


**Figure S8.1:26.** Reference standard chromatogram and MS^2^ spectrum of 2-chloro-4-nitroaniline.

### **S8.2** Non-target screening


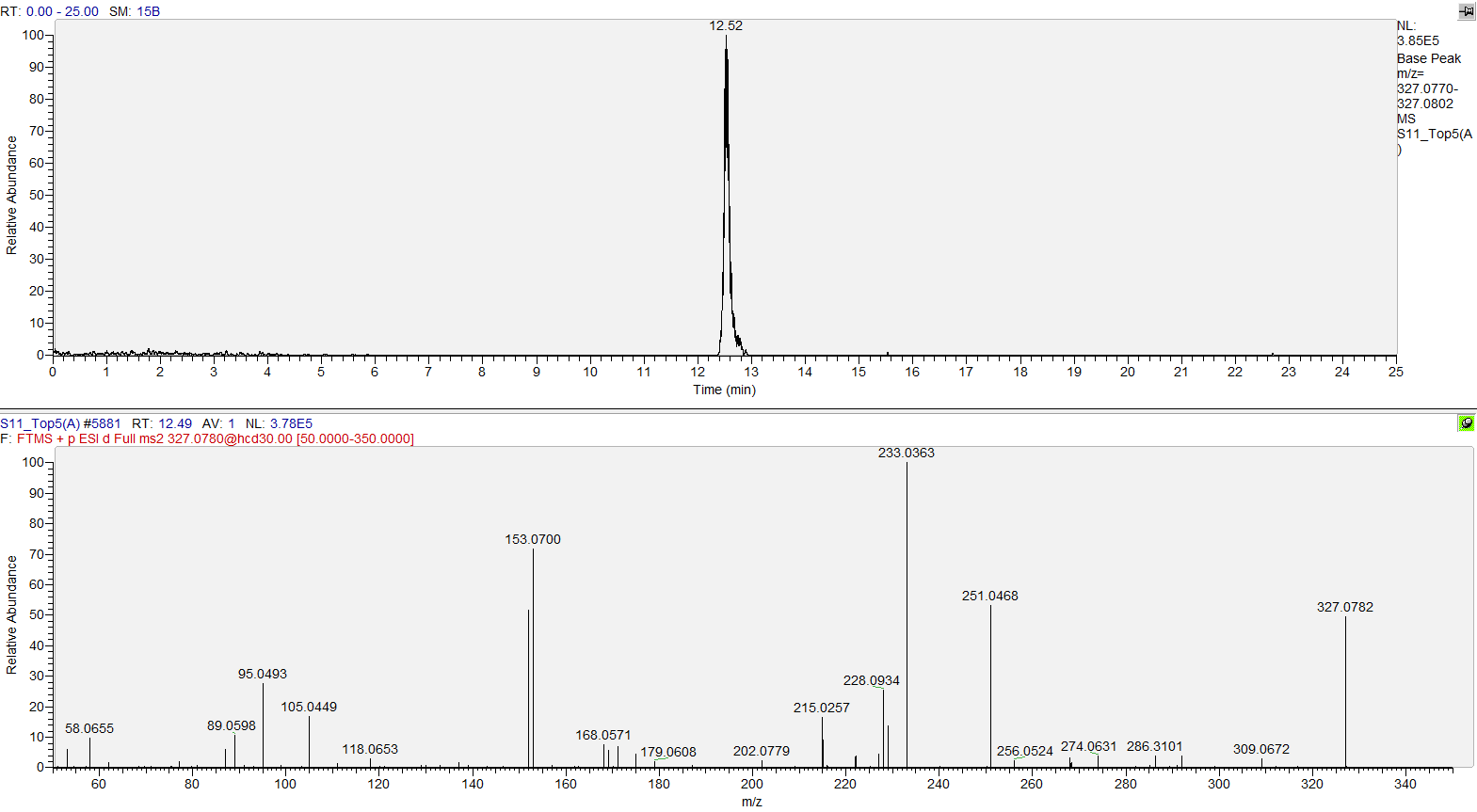


**Figure S8.2:1.** Chromatogram and MS^2^ spectrum of triphenyl phosphate (TPP) in sample 11.


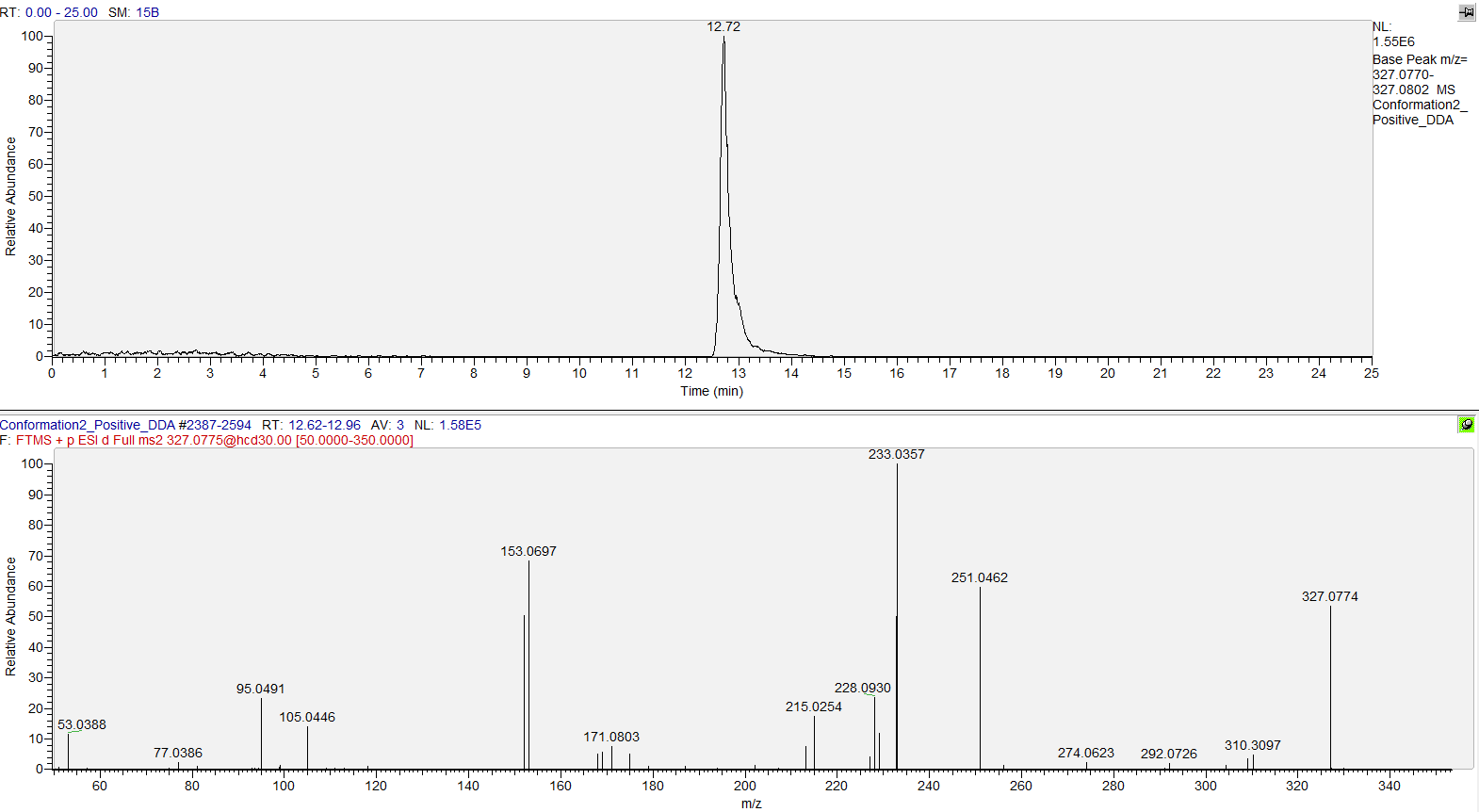


**Figure S8.2:2.** Reference standard chromatogram and MS^2^ spectrum of triphenyl phosphate (TPP).


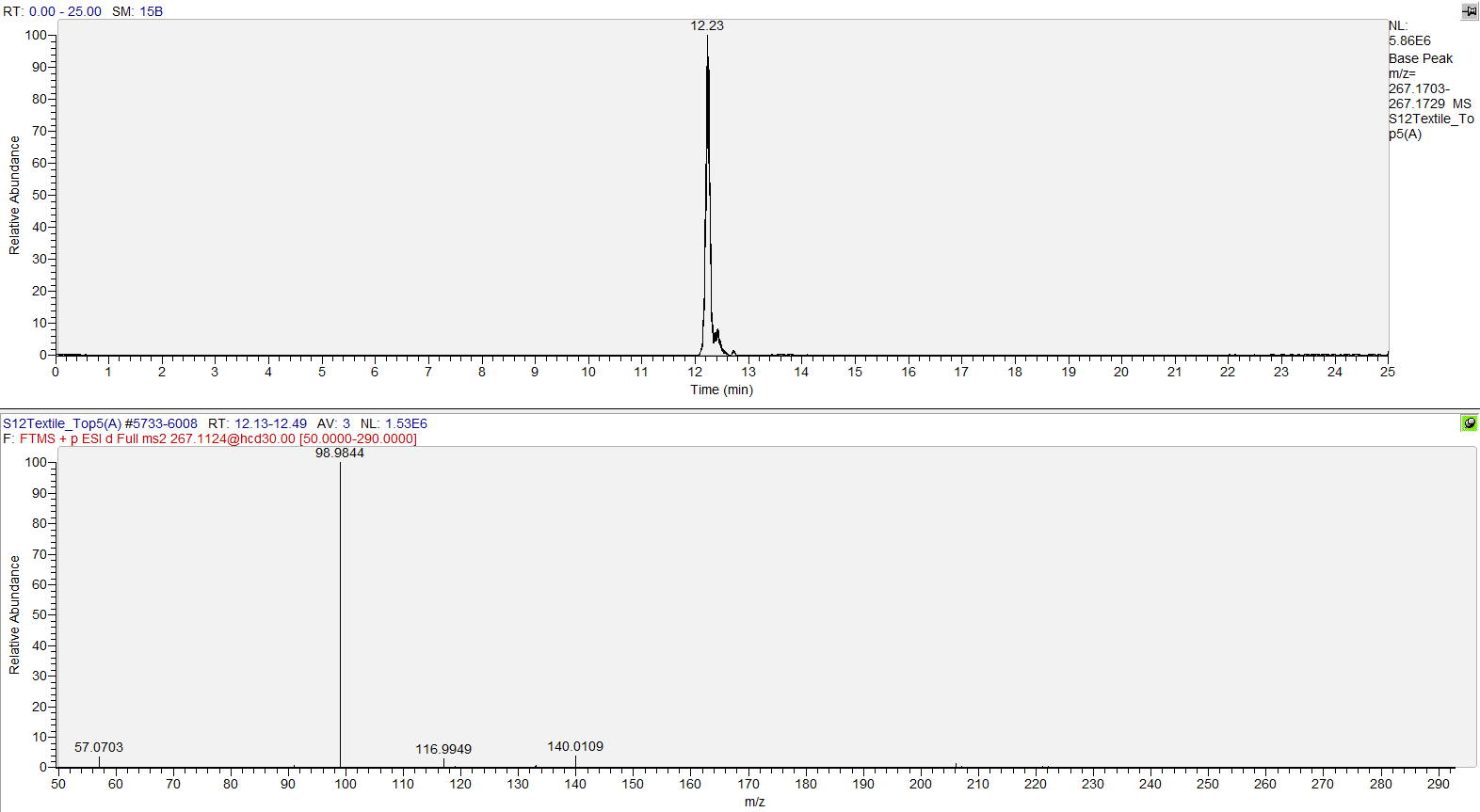


**Figure S8.2:3.** Chromatogram and MS^2^ spectrum of tributyl phosphate (TBP) in sample 12.


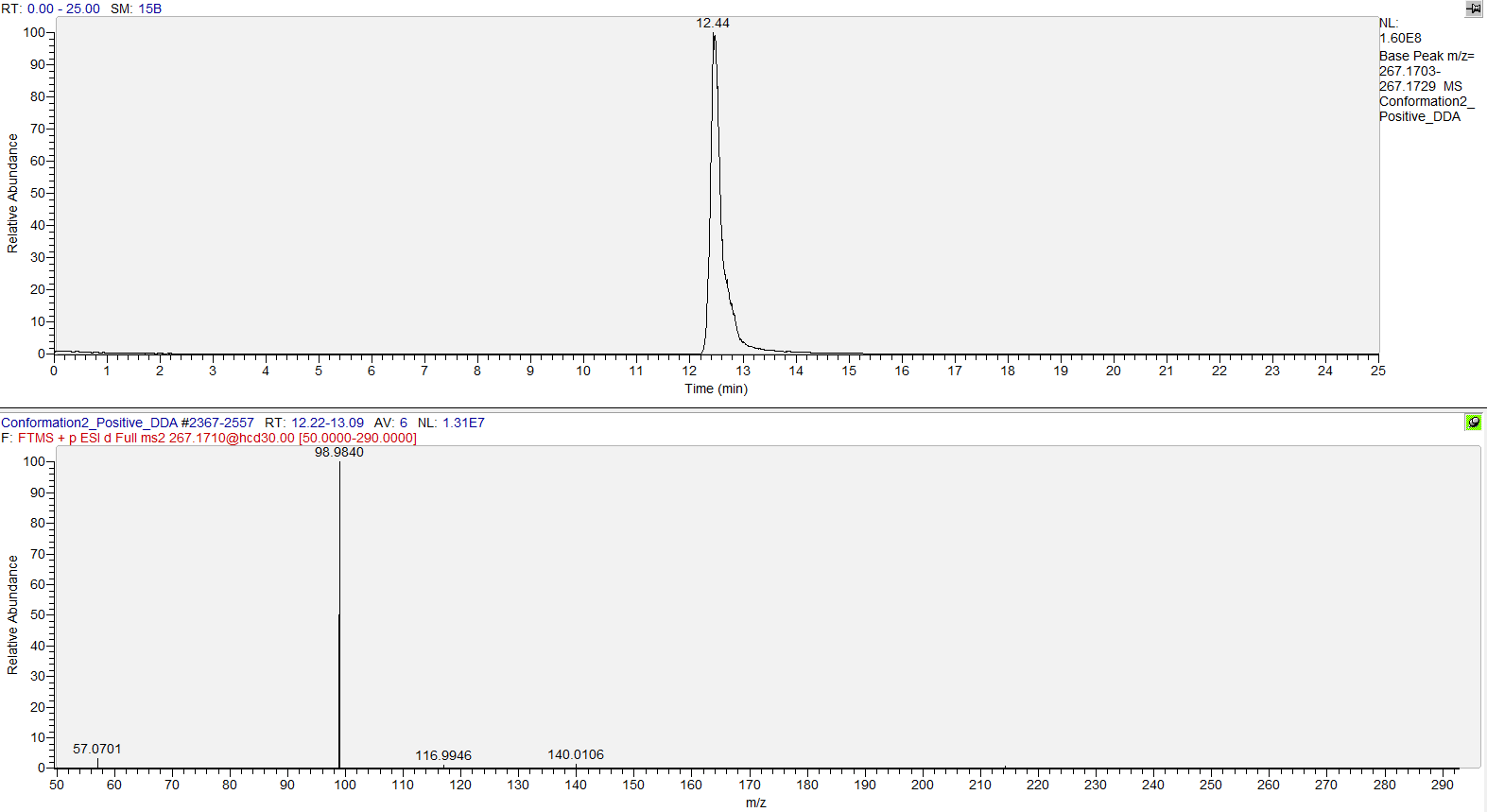


**Figure S8.2:4.** Reference standard chromatogram and MS^2^ spectrum of tributyl phosphate (TBP).


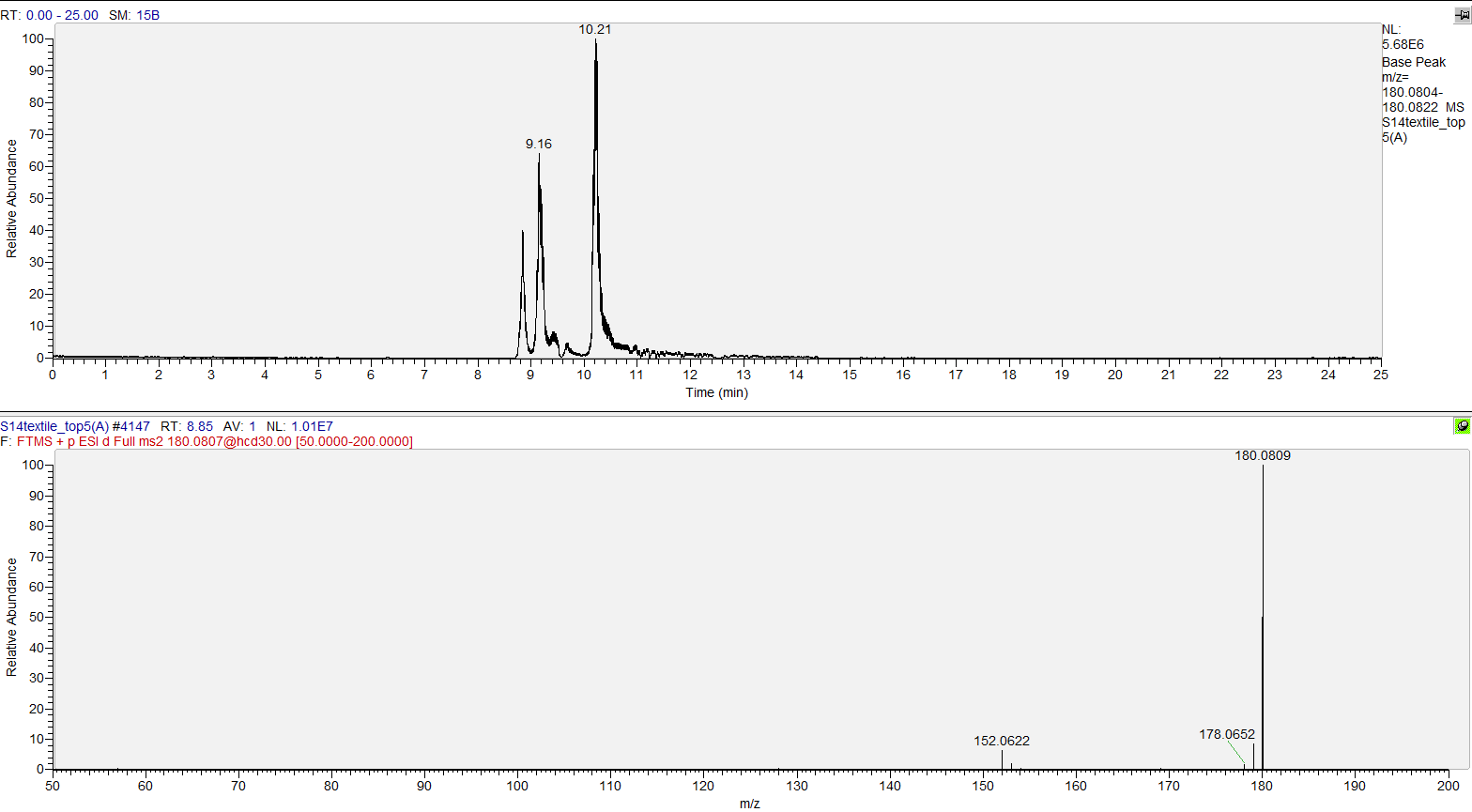


**Figure S8.2:5.** Chromatogram and MS^2^ spectrum of acridine in sample 14.


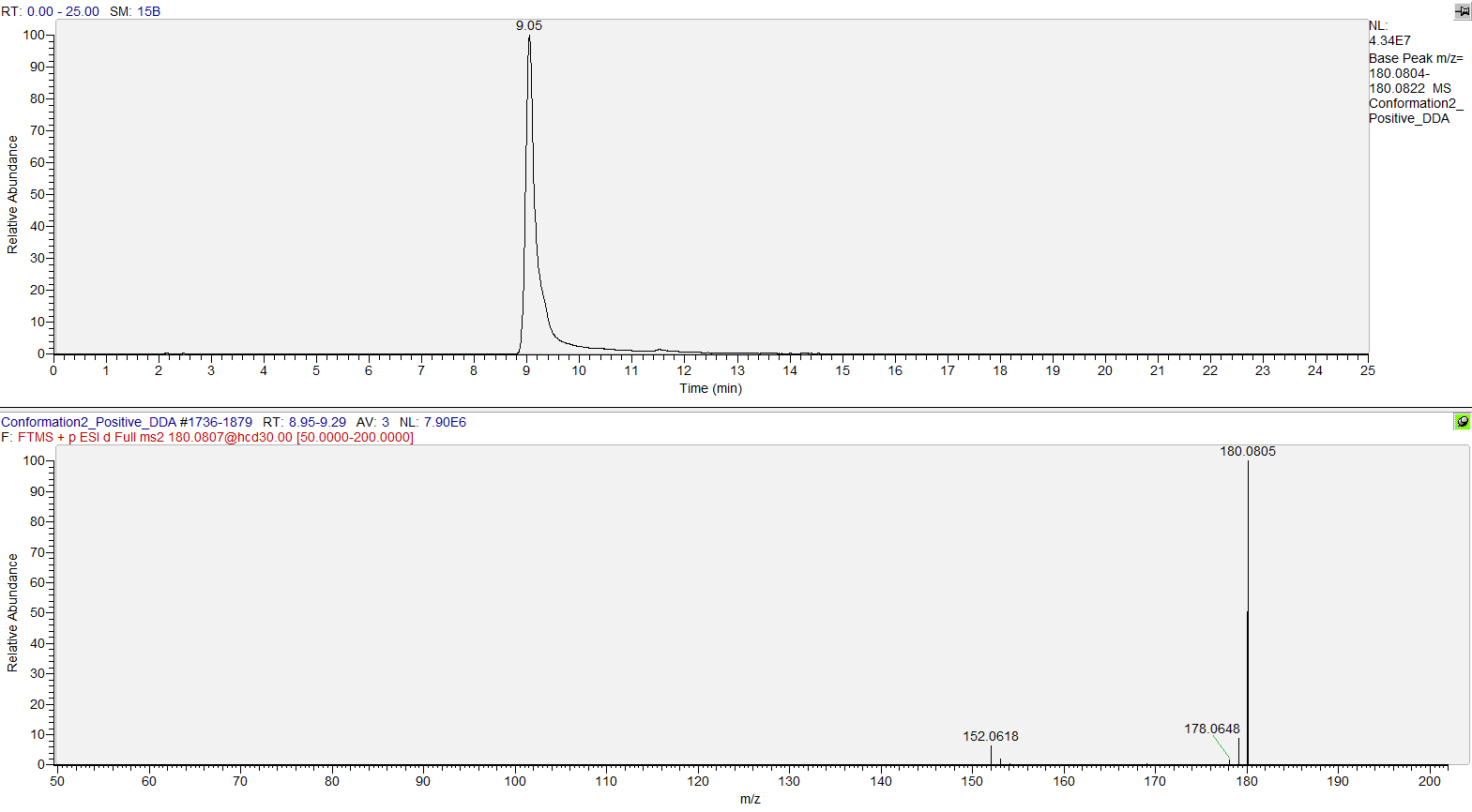


**Figure S8.2:6.** Reference standard chromatogram and MS^2^ spectrum of acridine.


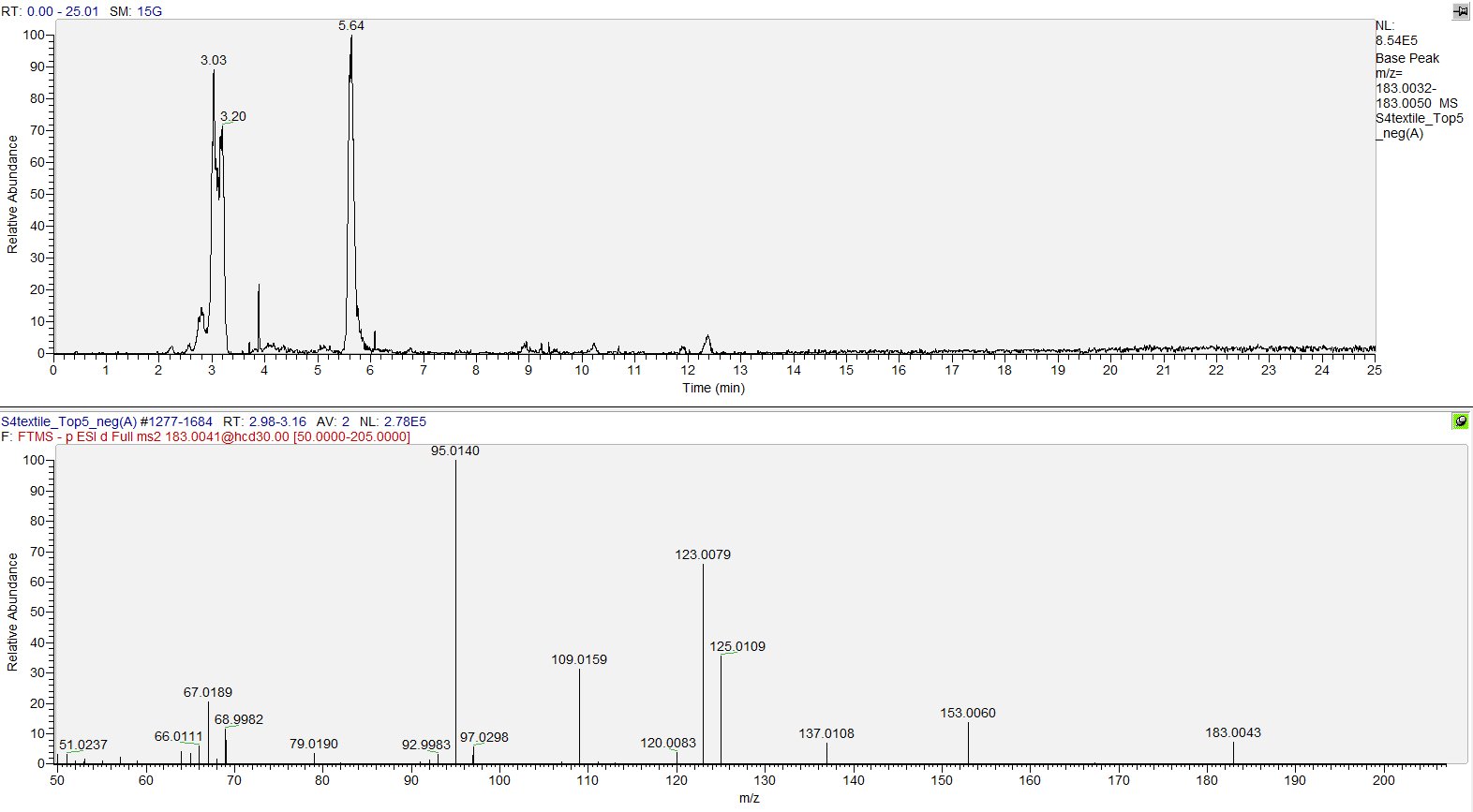


**Figure S8.2:7.** Chromatogram and MS^2^ spectrum of 2,4-dinitrophenol in sample 4.


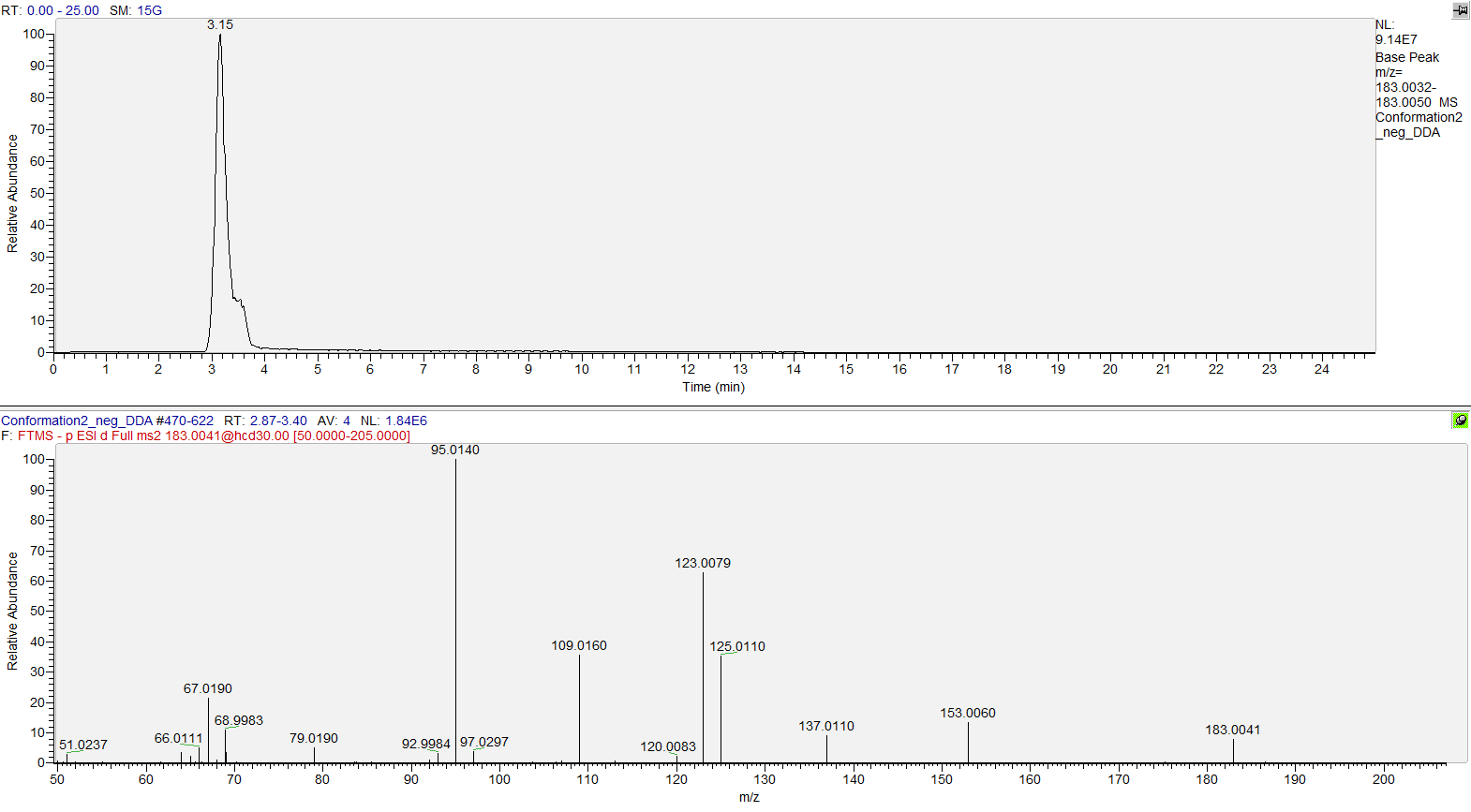


**Figure S8.2:8.** Reference standard chromatogram and MS^2^ spectrum of 2,4-dinitrophenol.


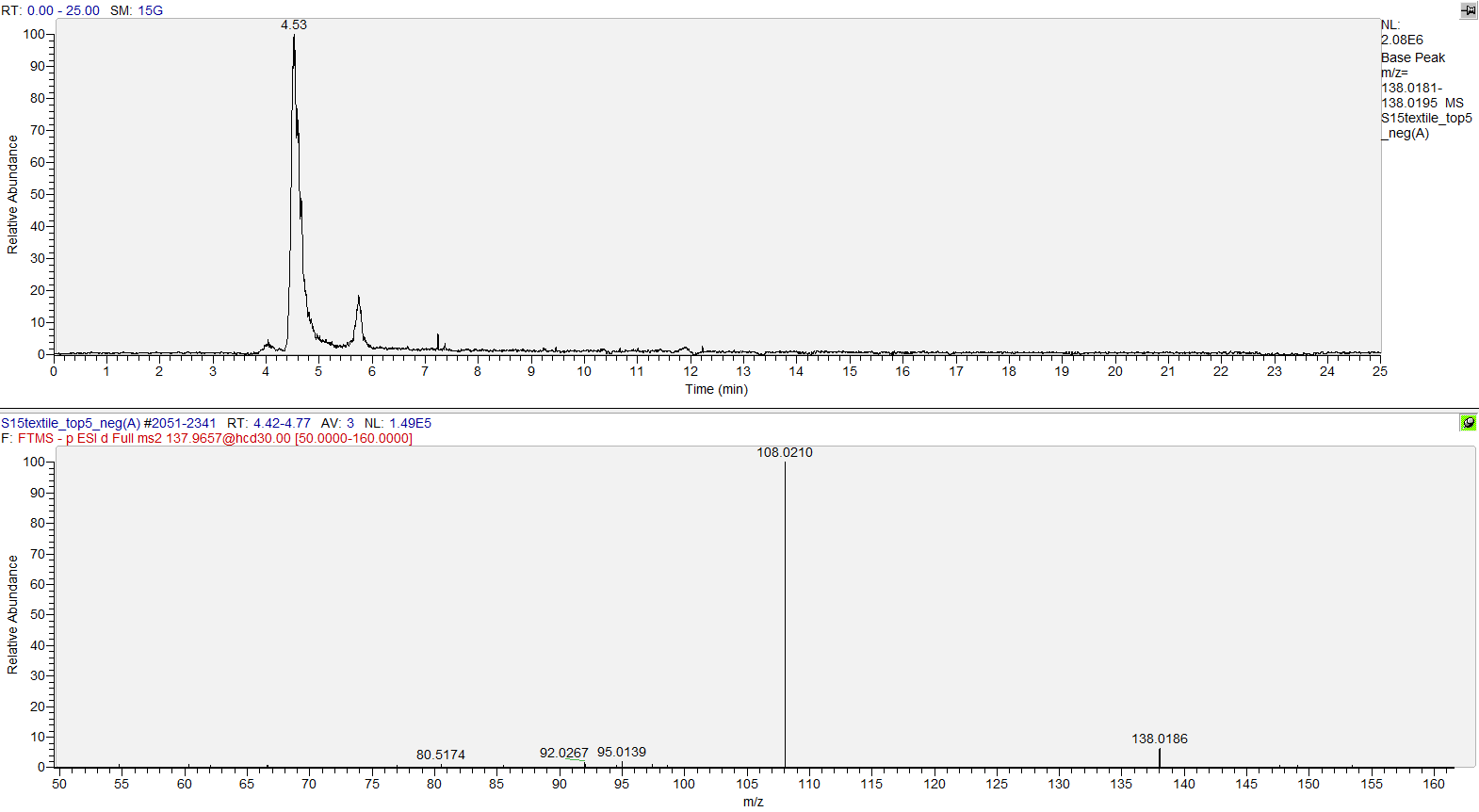


**Figure S8.2:9.** Chromatogram and MS^2^ spectrum of 4-nitrophenol in sample 15.


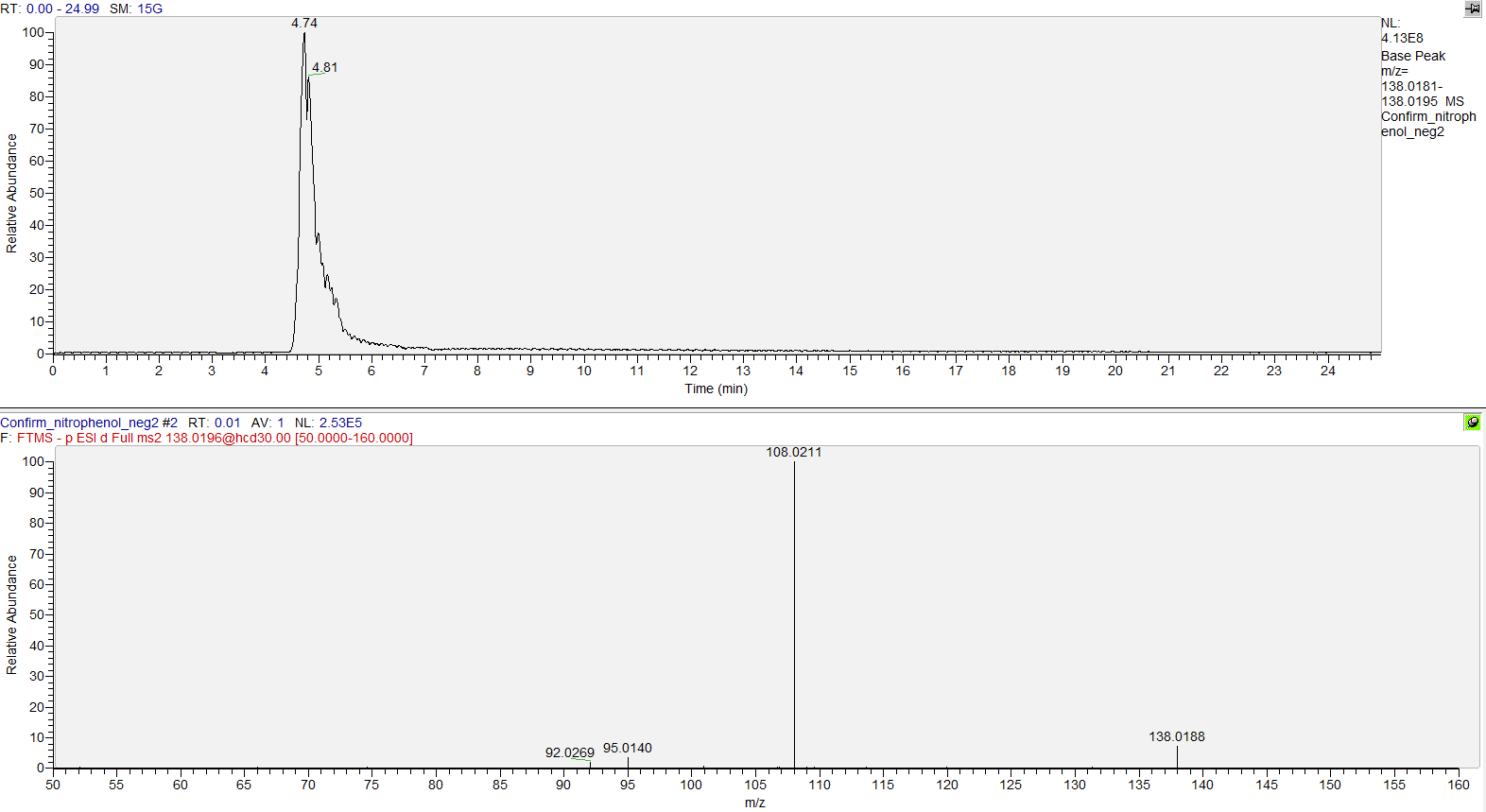


**Figure S8.2:10.** Reference standard chromatogram and MS^2^ spectrum of 4-nitrophenol.


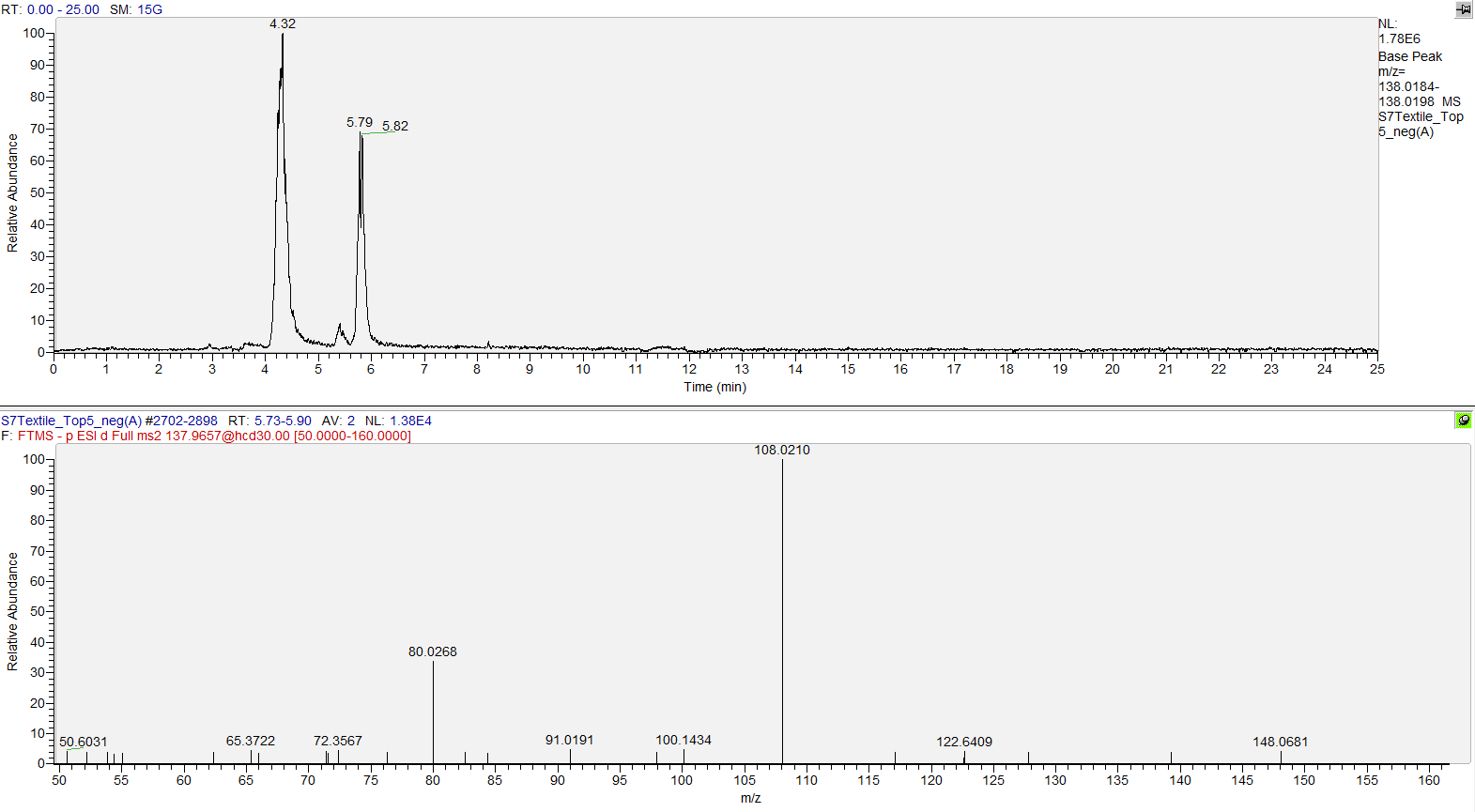


**Figure S8.2:11.** Chromatogram and MS^2^ spectrum of 3-nitrophenol in sample 7.


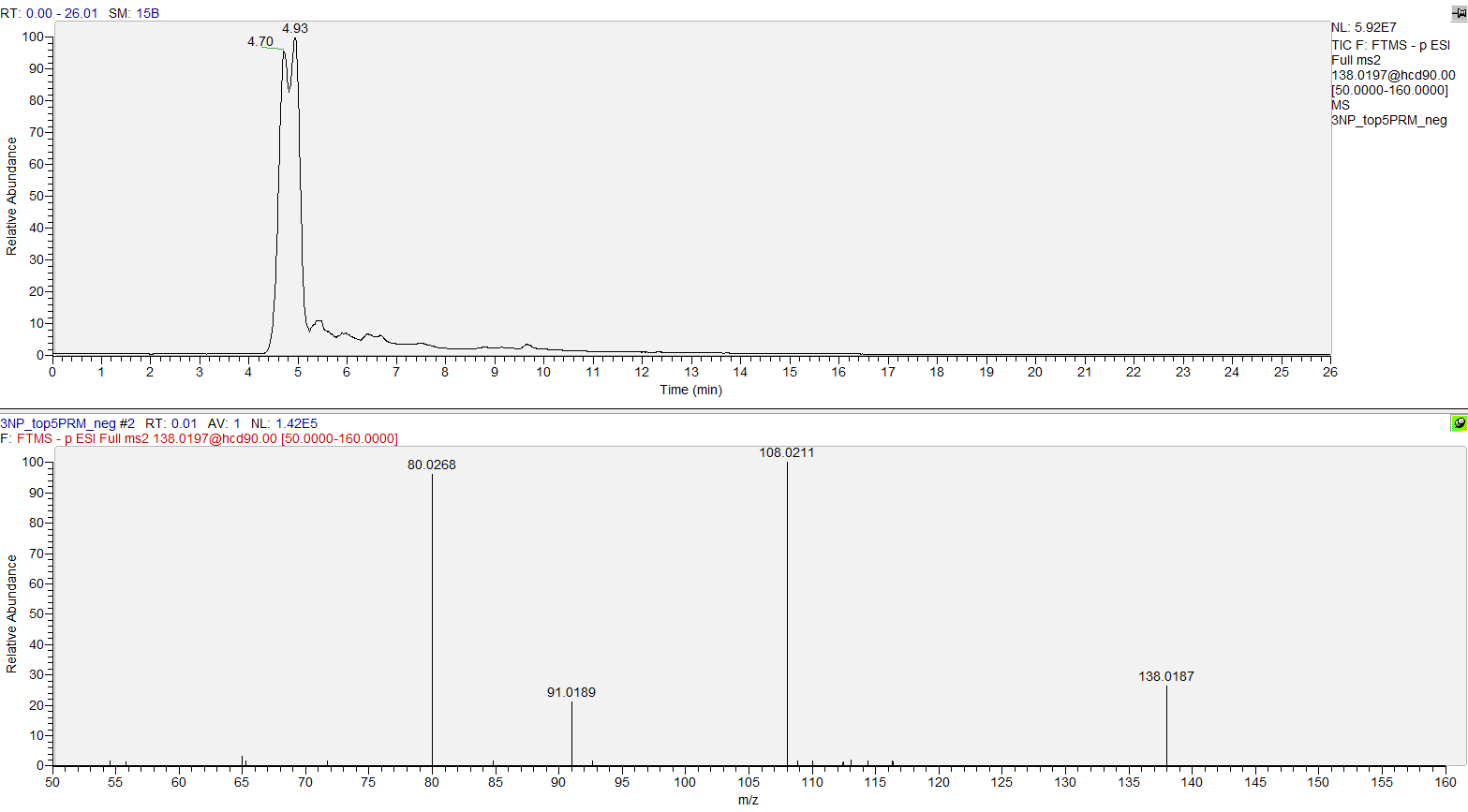


**Figure S8.2:12.** Reference standard chromatogram and MS^2^ spectrum of 3-nitrophenol.


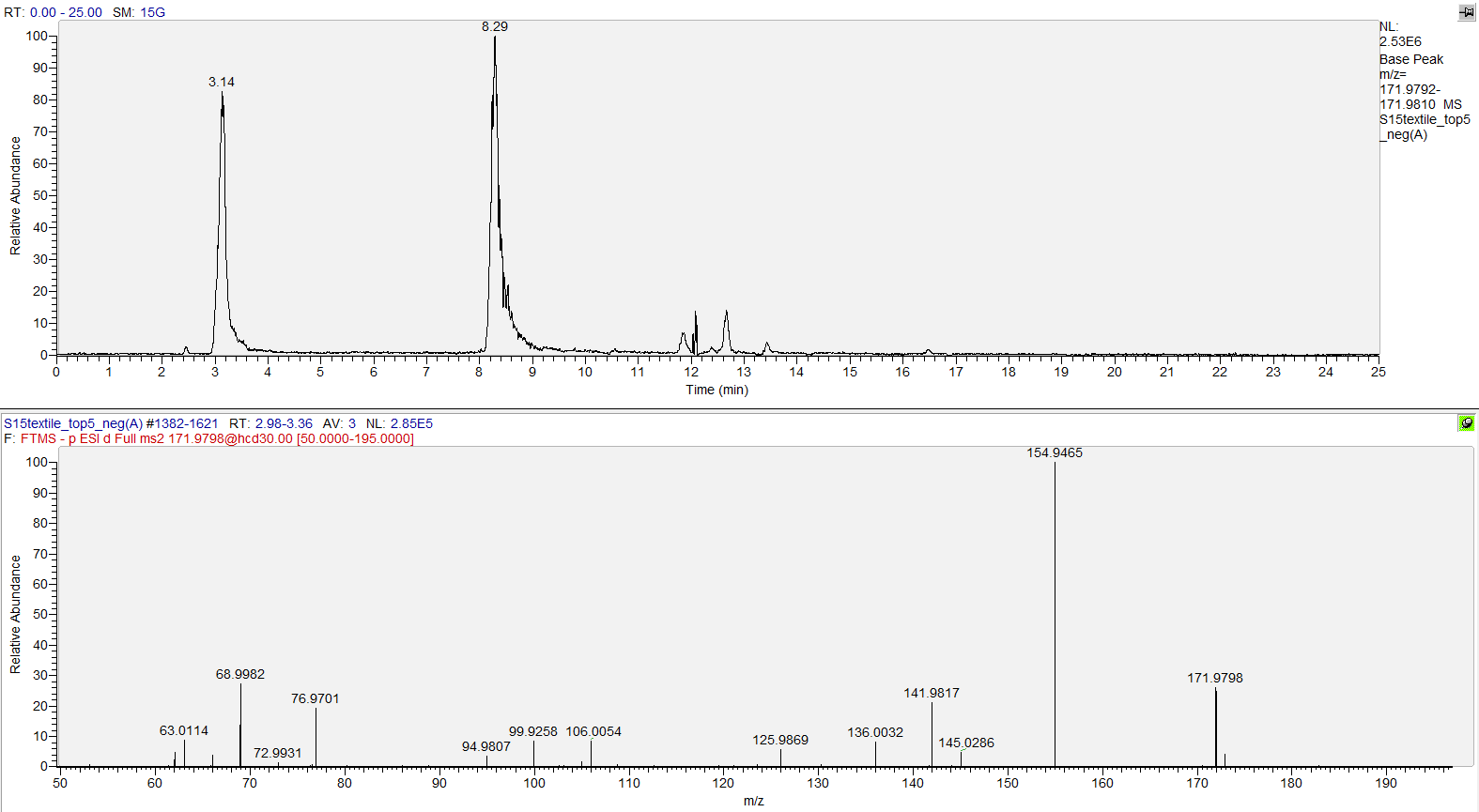


**Figure S8.2:13.** Chromatogram and MS^2^ spectrum of x-chloro-y-nitrophenol in sample 15.


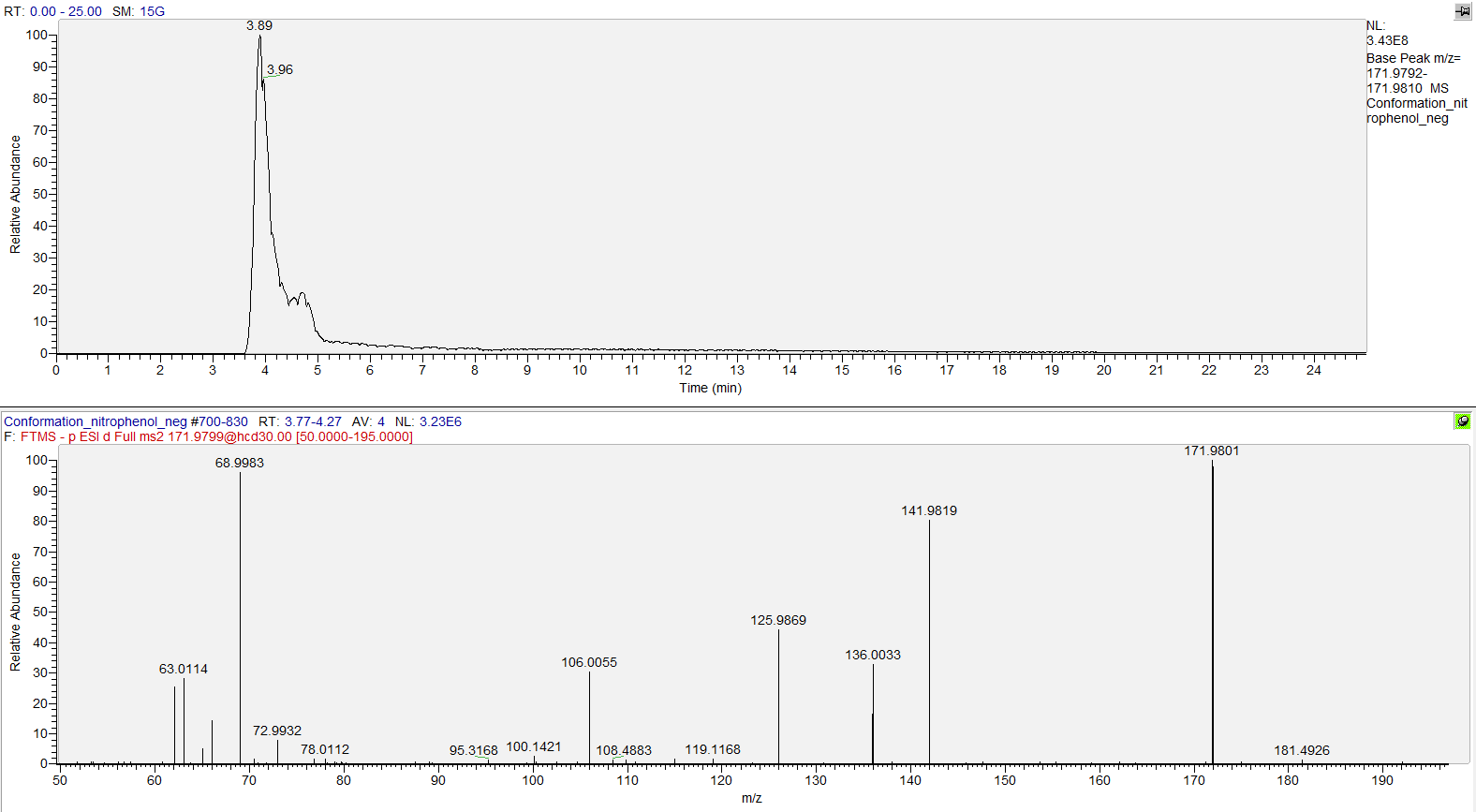


**Figure S8.2:14.** Reference standard chromatogram and MS^2^ spectrum of 2-chloro-4-nitrophenol.
